# FOXM1 regulates glycolysis and energy production in multiple myeloma

**DOI:** 10.1038/s41388-022-02398-4

**Published:** 2022-07-06

**Authors:** Yan Cheng, Fumou Sun, Krista Thornton, Xuefang Jing, Jing Dong, Grant Yun, Michael Pisano, Fenghuang Zhan, Sung Hoon Kim, John A. Katzenellenbogen, Benita S. Katzenellenbogen, Parameswaran Hari, Siegfried Janz

**Affiliations:** 1 Division of Hematology and Oncology, Department of Medicine, Medical College of Wisconsin, Milwaukee, WI USA; 2 Department of Pathology Master of Science Graduate Program, University of Iowa, Iowa City, IA USA; 3 Department of Pathology, University of Iowa, Iowa City, IA USA; 4 Interdisciplinary Graduate Program in Immunology, University of Iowa, Iowa City, IA USA; 5 Myeloma Center, Division of Hematology and Oncology, Department of Medicine, and Winthrop P. Rockefeller Cancer Institute, University of Arkansas for Medical Sciences, Little Rock, AR USA; 6 Departments of Chemistry, University of Illinois at Urbana-Champaign, Urbana, IL USA; 7 Molecular and Integrative Physiology, and Cancer Center at Illinois, University of Illinois at Urbana-Champaign, Urbana, IL USA; 8 Medical College of Wisconsin Cancer Center, Milwaukee, WI USA

**Keywords:** Cancer metabolism, Myeloma, Translation, Prognostic markers

## Abstract

The transcription factor, forkhead box M1 (FOXM1), has been implicated in the natural history and outcome of newly diagnosed high-risk myeloma (HRMM) and relapsed/refractory myeloma (RRMM), but the mechanism with which FOXM1 promotes the growth of neoplastic plasma cells is poorly understood. Here we show that FOXM1 is a positive regulator of myeloma metabolism that greatly impacts the bioenergetic pathways of glycolysis and oxidative phosphorylation (OxPhos). Using FOXM1-deficient myeloma cells as principal experimental model system, we find that FOXM1 increases glucose uptake, lactate output, and oxygen consumption in myeloma. We demonstrate that the novel 1,1-diarylethylene small-compound FOXM1 inhibitor, NB73, suppresses myeloma in cell culture and human-in-mouse xenografts using a mechanism that includes enhanced proteasomal FOXM1 degradation. Consistent with the FOXM1-stabilizing chaperone function of heat shock protein 90 (HSP90), the HSP90 inhibitor, geldanamycin, collaborates with NB73 in slowing down myeloma. These findings define FOXM1 as a key driver of myeloma metabolism and underscore the feasibility of targeting FOXM1 for new approaches to myeloma therapy and prevention.

## Introduction

FOXM1 is a promising molecular target in newly diagnosed high-risk myeloma (HRMM) [1] and relapsed/refractory myeloma (RRMM) [2, 3], but the mechanism with which FOXM1 promotes plasma cell neoplasia is poorly understood. FOXM1 is a transcription factor from the forkhead box family of proteins (n ≅ 50) characterized by a conserved winged helix DNA binding domain. FOXM1 is a validated oncoprotein in solid and liquid cancers [4] and governs, in part, the maintenance of cancer stem cell-like cells in breast cancer [5]. Whether FOXM1 is similarly important for the putative myeloma stem cell is not known as the elusiveness of this cell stands in the way of resolving the issue [6]. Recent large-scale pan-cancer analyses have shown that overexpression of FOXM1 is common in cancer and tightly associated with dismal outcome [7]. Although this has augmented efforts to target FOXM1 therapeutically [8], FOXM1 inhibitors are not yet available in the clinic. Here, we demonstrate that the novel small-compound FOXM1 inhibitor NB73 [9, 10] binds and destabilizes FOXM1 in myeloma cells and suppresses myeloma in cell culture and human-in-mouse xenografts. We show that FOXM1 is a positive regulator of myeloma metabolism with major impact on the bioenergetic pathways of glycolysis and oxidative phosphorylation (OxPhos). Relying on CRISPR/Cas9 editing as research tool, we provide definitive genetic evidence that, even though FOXM1 is not essential for growth and survival of neoplastic plasma cells, it upregulates glycolytic ATP production and promotes aerobic glycolysis (Warburg effect). Inspired by findings that heat shock protein 90 (HSP90) physically interacts with and stabilizes FOXM1 in myeloma cells, we show that HSP90 inhibitor, geldanamycin (GDA), collaborates with NB73 in slowing down myeloma. These findings further our understanding of the mechanism with which FOXM1 drives myeloma and support the feasibility of targeting FOXM1 pharmacologically for cancer therapy and prevention.

## Results

### Elevated *FOXM1* expression predicts poor survival in MMRF CoMMpass study

**Fig. 1:**
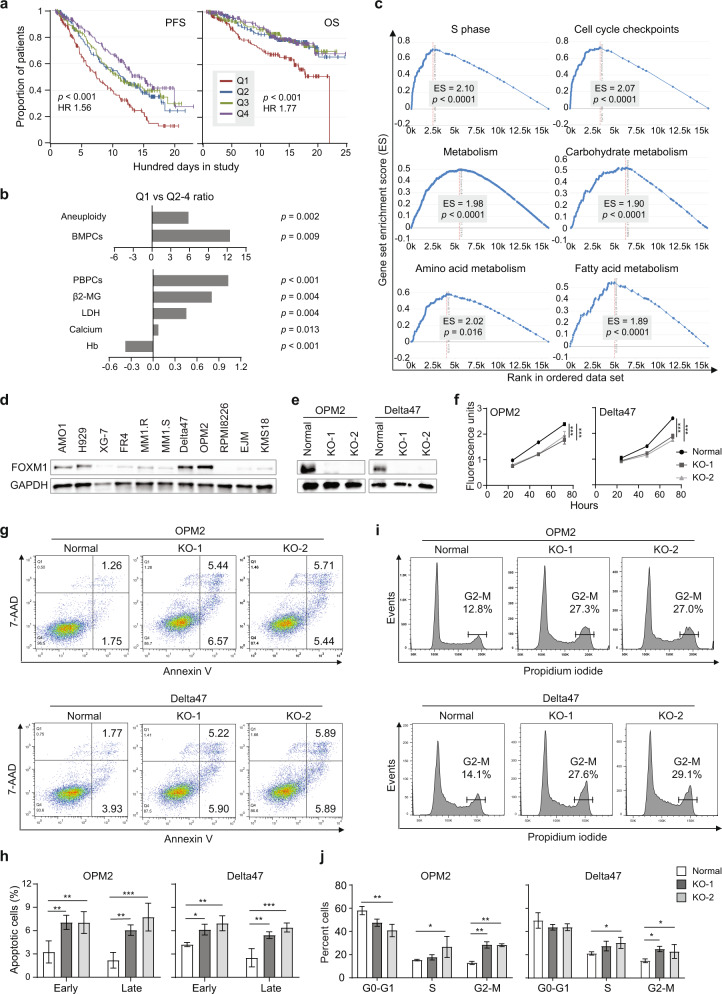
While elevated *FOXM1* message predicts poor survival of patients with myeloma, loss of FOXM1 inhibits myeloma in vitro. **a** Kaplan–Meier plot of progression free survival (PFS) and overall survival (OS) of patients with myeloma from the Multiple Myeloma Research Foundation (MMRF) CoMMpass trial (*n* = 773), divided into 4 quartiles (*n* = 193 in Q1-3, *n* = 194 in Q4) according to *FOXM1* mRNA levels in tumor cells. **b** Bar diagram indicating the increased severity of clinical features of myeloma in CoMMpass patients with top levels of *FOXM1* mRNA in the first quartile (Q1) compared to patients in the 2nd, 3rd and 4th quartile (Q2-4). The Q1 to Q2-4 ratios are plotted. Q1 patients exhibited significantly increased numbers of both plasma cells circulating in the peripheral blood (PBPCs) and tumor cells containing abnormal number of chromosomes (aneuploidy), elevated blood serum levels of β2-microglobulin (β2-MG), lactate dehydrogenase (LDH) and calcium, and reduced levels of hemoglobulin (Hb). **c** Gene set enrichment analysis (GSEA) plots using median *FOXM1* expression in the CoMMpass dataset as cutoff. Compared to *FOXM1*^Low^ myeloma (Q3-4 in (**a**)), *FOXM1*^High^ myeloma (Q1-2) features significant enrichments in genetic networks that regulate cell cycle progression (top panels) or cell metabolism (center and bottom panels). **d** Western analysis of FOXM1 in 11 human myeloma cell lines (HMCLs). GAPDH was used as loading control. **e** Western blots of FOXM1 and GAPDH in 2 independent, FOXM1-deficient OPM2 and Delta47 daughter cell lines (labeled as “knock outs” KO-1 and KO-2) compared to FOXM1-proficient parental cells (control). **f** Growth curves of OPM2 and Delta47 cells distinguished by proficiency (black) or deficiency (light and dark gray) for FOXM1. **g** Representative flow cytometric scatter plots of programmed cell death (apoptosis) of FOXM1 proficient or deficient OPM2 cells (top) and Delta47 cells (bottom). Percent cells undergoing early (Annexin^+^AAD^-^) and late (Annexin^+^AAD^+^) stages of apoptosis are indicated. **h** Mean values (vertical bars) and standard deviations (short horizontal lines) of OPM2 cells (left) and Delta47 cells (right) in early or late apoptosis based on triplicate measurements. **i** Representative flow cytometric histograms of cell cycle progression of FOXM1 proficient or deficient OPM2 cells (top) and Delta47 cells (bottom). **j** Mean values and standard deviations of OPM2 and Delta47 cells in three different stages of the cell cycle based on triplicate measurements: G, growth phase; S, synthesis phase, M, mitotic phase.

We sought to determine whether upregulation of *FOXM1* may be a prognosticator of dismal survival in the Multiple Myeloma Research Foundation (MMRF) CoMMpass study (NCT01454297). We chose this study because it evaluates myeloma progression in over one thousand patients in a comprehensive, publicly accessible, longitudinal, and prospective fashion [11, 12], and thereby provides an unequaled platform for clinical and outcome studies (for a recent update, see Skerget et al. at The Preprint Server for Health Sciences, medRxiv: 10.1101/2021.08.02.21261211). Kaplan–Meier analysis of CoMMpass patients for which *FOXM1* expression data were available (*n* = 773) demonstrated a significant reduction in progression free survival (PFS) and overall survival (OS) in the top quartile (Q1) exhibiting peak levels of *FOXM1* mRNA in neoplastic plasma cells (Fig. 1a). Cox proportional hazard analysis showed that *FOXM1* expression is an independent survival factor in this dataset (Table S1). Moreover, elevation of *FOXM1* message was associated with elevated expression of *MYC* (Fig. S1) and statistical comparison of paired baseline and progression samples from 65 patients revealed that *FOXM1* underwent, on average, a ~2-fold upregulation during tumor progression (Fig. S2). These results validated previous findings on heightened expression of *FOXM1* in subsets of HRMM [1] and drug resistant RRMM [2, 3]. Elevated *FOXM1* expression was strongly associated with increased severity of clinical parameters, such as increased abundance of plasma cells in bone marrow and peripheral blood, heightened tumor cell aneuploidy, increased serum levels of β2-microglobulin, lactate dehydrogenase and calcium and, last but not least, decreased amounts of hemoglobin, the hallmark of anemia (Fig. 1b, Fig. S3). Next, we employed global RNA-seq expression profiles from the CoMMpass dataset to determine differentially expressed genes (DEGs) in tumors with high (Q1-2) and low (Q3-4) *FOXM1* message levels. Gene set enrichment analysis (GSEA) of DEGs not only demonstrated activation of cell cycle progression pathways such as *S phase* and *Cell cycle checkpoints* in *FOXM1* high (FOXM1^High^) myeloma (Fig. 1c, top) but also pointed to broad upregulation of metabolic pathways including *Metabolism*, *Carbohydrate*, *Amino acids and derivatives* and *Fatty acids, Triacylglycerol and Ketone bodies* (Fig. 1c, center and bottom). These findings were consistent with the well-established role of *FOXM1* as an oncogene that drives cancer by promoting cell proliferation [13] and further suggested that *FOXM1* is an important regulator of myeloma metabolism.

### FOXM1 is not essential in myeloma but promotes its growth and survival

**Fig. 2:**
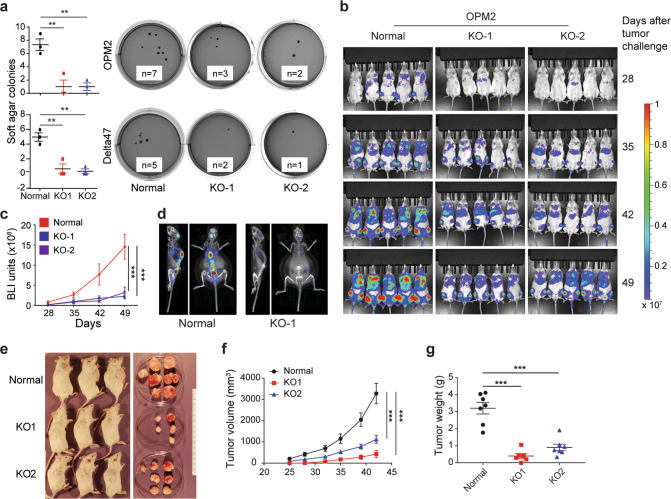
Loss of FOXM1 inhibits clonogenic myeloma growth in soft agar and slows down human-in-mouse xenografts. **a** Scatter plot comparing mean values (long horizontal lines) and standard deviations (short horizontal lines) of soft-agar colonies of FOXM1-proficient (control) and FOXM1-deficient (KO-1 and KO-2) OPM2 cells (top panel) and Delta47 cells (bottom). Representative photographic images of soft-agar plates containing clonal outgrowths of myeloma cells (black dots) are shown to the right. The number of colonies above a predefined size is indicated. **b** Sequential BLI images of NSG mice carrying FOXM1-proficient (control) or FOXM1-deficient (KO) OPM2 myeloma xenografts. **c** Mean BLI intensities of mice shown in (**b**). Standard deviation of the mean is indicated by vertical lines. **d** BLI and CT fusion images of 2 different NSG mice that harbor a xenografted FOXM1^N^ (left) or FOXM1^KO^ (right) OPM2 myeloma. **e** Photographic images of NSG mice carrying SC xenografts of OPM2 tumors that are either FOXM1^N^ (top row) or FOXM1^KO^ (center and bottom rows). During necropsy of mice on day 42 after tumor cell challenge, the tumors were excised in toto and placed in a petri dish for visual inspection. **f** Growth rate of tumors shown in panel e. Tumor diameters were measured on indicated days following tumor propagation. Tumor volumes were calculated and plotted. Shown are mean values (*n* = 3) and SDs (short vertical lines). **g** Weight of tumors shown in panel e.

The myeloma-promoting role of FOXM1 has been documented [1, 2], but whether FOXM1 is essential in myeloma has not yet been established. To address this knowledge gap, we used Western blotting to measure FOXM1 protein levels in 11 human myeloma cell lines (HMCLs) (Fig. 1d). We chose the two lines that expressed the largest amount of the transcription factor, OPM2 and Delta47, for gene editing effected by CRISPR-Cas9. In both cases, two independent FOXM1-deficient daughter lines, designated KO-1 and KO-2, were generated (Fig. 1e). They contained 2 different 10-bp deletions at the target site of their respective guide RNA in case of OPM2 and a 10-bp deletion and 1-bp insertion in case of Delta47 (Fig. S4). Western analysis confirmed the lack of FOXM1 in all “knockout” clones, referred to hereafter as FOXM1^KO^. FOXM1-deficient myeloma cells proliferated more slowly than their parental counterparts that contained normal levels of FOXM1, henceforth called FOXM1^N^ and used as control (Fig. 1f). Flow cytometric measurements of programmed cell death after staining with 7-aminoactinomycin D (AAD) and antibody to annexin A5 (AV), showed increased rates of early apoptosis (AV^+^AAD^-^) and late apoptosis (AV^+^AAD^+^) in FOXM1^KO^ cells compared to FOXM1^N^ cells (Fig. 1g). FOXM1-dependent loss of survival was somewhat higher in OPM2 than Delta47 cells (Fig. 1h). Flow cytometric analysis of cell cycle progression using propidium iodide (Fig. 1i) demonstrated a higher fraction of FOXM1^KO^ cells in G2-M relative to FOXM1^N^ cells (Fig. 1j). The proliferation and survival enhancing effect of FOXM1 was also evident in assessments of clonogenic myeloma growth in soft agar and progression of intravenous myeloma cell xenografts in NSG mice. Thus, FOXM1^KO^ OPM2 (top) and Delta47 (bottom) produced fewer colonies than their FOXM1^N^ counterparts in colony formation assays (Fig. 2a). In agreement with that, clonogenicity increased upon forced expression of FOXM1 in MM1.S myeloma cells (Fig. S5) that contain low amounts of the transcription factor (Fig. 1d, lane 6). OPM2 FOXM1^KO^ lagged behind FOXM1^N^ in human-in-mouse xenografts, using serial whole-body bioluminescence imaging (BLI, Fig. 2b) as unbiased measurement tool (Fig. 2c). Integrated BLI and computed tomography (CT) scans of xenografted NSG mice demonstrated reduced bone marrow involvement of FOXM1^KO^ OPM2 cells (Fig. 2d, right) compared to FOXM1^N^ cells. The latter exhibited marked proclivity to home to the central skeleton (Fig. 2d, left), a distinct advantage of this cell line compared to other HMCLs. Diminished growth of OPM2 FOXM1^KO^ cells relative to FOXM1^N^ cells was also seen upon subcutaneous tumor propagation (Fig. 2e), with growth rate measurements based on tumor volume (Fig. 2f) and tumor weight (Fig. 2g) as outcome metrics. These results showed that – even though FOXM1 plays an important role in growth and survival of myeloma—it is not essential. This is consistent with published work that genetic deletion of FOXM1 in hematopoietic stem cells [14] and early B-cell progenitors [15] is compatible with B cell and plasma cell development.

### FOXM1-dependent gene expression changes in myeloma

**Fig. 3:**
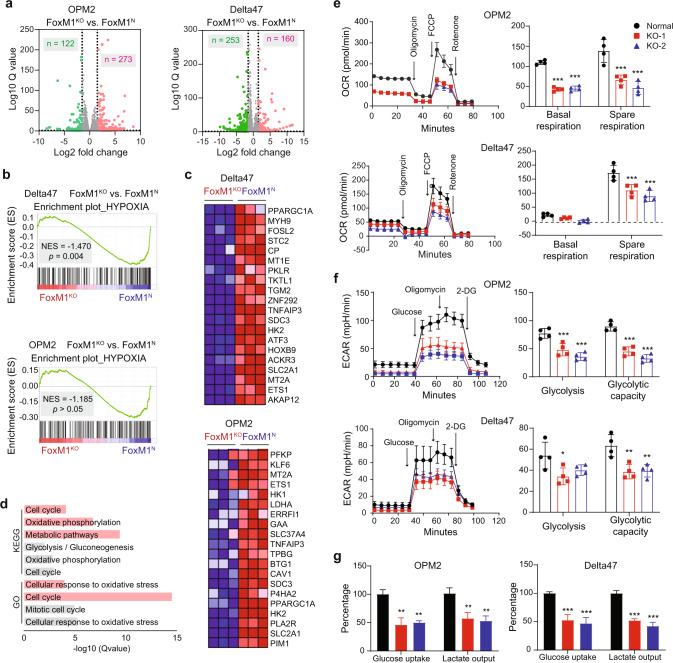
FOXM1-dependent gene expression, glycolysis and energy production. **a** Volcano plot of differentially expressed genes (DEGs) in FOXM1^N^ vs FOXM1^KO^ OPM2 cells (left) and Delta47 cells (right). Up and down regulated genes are indicated in red and green, respectively. **b** GSEA plots of DEGs from panel (**a**). OPM2, top; Delta47, bottom. **c** Heatmap of top 20 genes in *Hypoxia* shown in (**b**). *SLC2A1* encodes GLUT1. **d** FOXM1-dependent KEGG and GO biological processes in OPM2 (gray) and Delta47 (red) cells. DEGs from panel a were used as input for the analysis. **e** Line diagram depicting the oxygen consumption rate (OCR) of OPM2 (top) and Delta47 (bottom) cells containing normal levels of FOXM1 (control) or lacking FOXM1 (KO-1 and KO-2). Steady-state baseline conditions in the first 30 min were interrupted by addition of metabolic modulators (indicated in left panels by arrows pointing down that are labeled) to determine base and spare respiration capacity (right panels). **f** Line diagram of extracellular acidification rate (ECAR) under baseline conditions (left panel) challenged by small compounds that permit the determination of glycolysis and glycolytic capacity (right panel). **g** Bar diagrams of mean glucose uptake (columns 1–3) and mean lactate secretion (columns 4–6) based on 3 independent measurements in FOXM1^KO^ cells (red and blue) compared to FOXM1^N^ cells (black).

Following up on the results in Fig. 1c, we wondered whether FOXM1-dependent gene expression changes in OPM2 and Delta47 would also point to metabolism as a central aspect of myeloma biology regulated by FOXM1. With that goal in mind, we employed genome-wide RNA sequencing to compare the transcriptional output of FOXM1^N^ and FOXM1^KO^ OPM2 and Delta47 cells. The results are summarized in volcano plots (Fig. 3a) using fold changes in gene expression of more than 1.5 log 2 (x axis) and statistical Q values of less than 0.05 (y axis) as cutoffs (dotted vertical lines) for differentially expressed genes (DEGs). Based on these parameters, OPM2 and Delta47 exhibited 393 DEGs (273 up and 122 down) and 413 DEGs (160 up and 252 down), respectively. GSEA revealed *Hypoxia* (Table S2) as a significantly enriched pathway in Delta47 (Fig. 3b, top). This pathway peaked our interest because it included *HK2* and *SLC2A1* among the top-20 differentially expressed pathway genes, not only in Delta47 (Fig. 3c, top) but also in OPM2 (Fig. 3c, bottom) even though the pathway as such was not significantly enriched in these cells (Fig. 3b, bottom). *HK2* encodes hexokinase 2, which catalyzes the first step of glycolysis – a key bioenergetic pathway in the cytoplasm that generates ATP by breaking down glucose into pyruvate. *SLC2A1* encodes *GLUT1*, which transports glucose from the extracellular milieu to the cytoplasm. The heatmap for OPM2 additionally contains *LDHA* (encoding lactate dehydrogenase A), which promotes growth [16] and drug resistance [17] of myeloma by virtue of lactate production. Analysis of DEGs using the KEGG (Kyoto Encyclopedia of Genes and Genomes) and GO (Gene Ontology) online tools (Fig. 3d) revealed that the genetic pathways of *Cellular response to oxidative stress* (GO) and *Cell cycle* and *Oxidative phosphorylation* (KEGG) were significantly involved in both OPM2 and Delta47 cells (Tables S3 and S4). GSEA also suggested involvement of a cell cycle-related pathway, dubbed G2M checkpoint, in both cell lines, although the evidence was stronger for Delta47 (Fig. S6). Just like glycolysis, OxPhos is a central pathway of energy production, generating ATP in the electron transport chain during mitochondrial respiration. To better appreciate the biological implications of the genomic findings, we used a Seahorse instrument to assess the role of FOXM1 in glycolysis and mitochondrial respiration. The results, which firmly implicated FOXM1 in the regulation of energy production in myeloma, will be described below.

### FOXM1 governs glycolysis and energy production in myeloma

Measurements of cellular respiration under steady-state conditions showed that the oxygen consumption rate (OCR) in OPM2 and Delta47 was reduced by ~50% in FOXM1^KO^ cells relative to FOXM1^N^ cells (Fig. 3e). Likewise, the spare reserve capacity in mitochondrial respiratory chain complexes was cut in half in FOXM1^KO^ cells compared to FOXM1^N^ cells (Fig. 3e). This capacity is the difference between basal and maximal respiratory rate and can be determined by sequential addition of oligomycin (inhibitor of complex V) and FCCP (proton-leaking uncoupling agent that disrupts ATP synthesis). Non-mitochondrial oxygen consumption, revealed by addition of rotenone (complex I inhibitor), was negligible regardless of FOXM1 status (Fig. 3e). To complement these findings with a measure of glycolytic activity, we determined the extracellular acidification rate (ECAR). It was significantly lower in FOXM1^KO^ cells compared to FOXM1^N^ cells under basal conditions (Fig. 3f). Peak glycolytic activity, which can be estimated upon sequential addition of excess substrate (glucose), oligomycin and 2-deoxyglucose (2-DG, competitive inhibitor of glycolysis), was also significantly reduced in FOXM1^KO^ cells relative to FOXM1^N^ cells (Fig. 3f). These results were supported by measurements of glucose uptake and lactate production, showing that both were reduced by ~50% in FOXM1^KO^ relative to FOXM1^N^ myeloma cells (Fig. 3g).

**Fig. 4:**
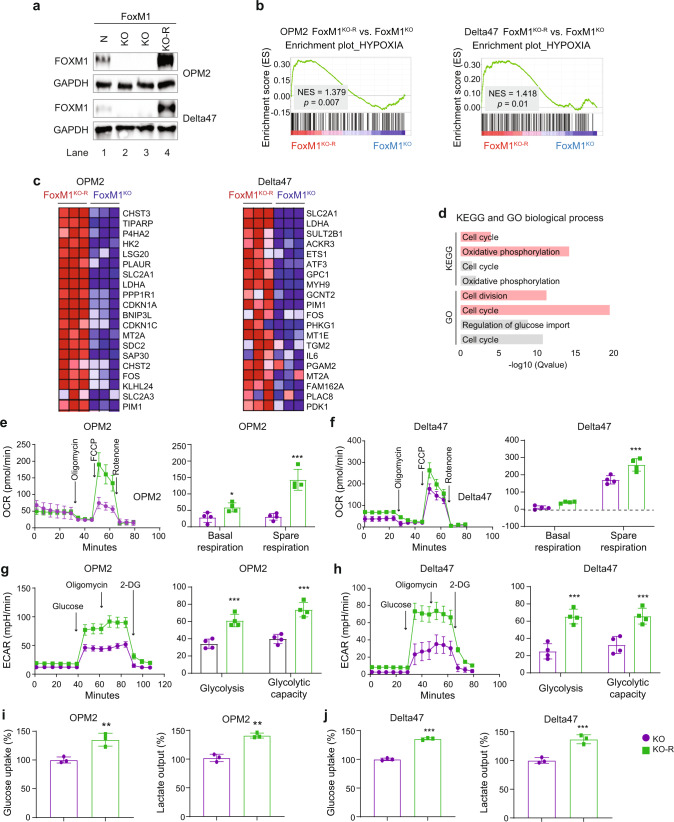
Gene expression and glycolytic activity in FOXM1 “add back” myeloma cells, FOXM1^KO-R^. **a** Western blot of FOXM1 levels in parental FOXM1^N^ cells (lane 1), derivative FOXM1^KO^ cells generated with the assistance of the CRISPR-Cas9 gene editing tool (lanes 2–3) and FOXM1-reconstituted FOXM1^KO-R^ cells generated by transfecting “knockout” cells with a constitutively expressed, lentivirus-encoded *FOXM1* cDNA gene. GAPDH, loading control; OPM2, top panel; Delta47, bottom panel. **b** Gene set enrichment plots of the *Hypoxia* genetic network based on differentially expressed genes (DEGs) in FOXM1^KO^ vs FOXM1^KO-R^ OPM2 (left) and Delta47 (right) cells. DEGs were determined, visualized in volcano plots analogous to Fig. 4a (not shown), and subjected to GSEA. NES, normalized enrichment score; FDR, false discovery rate. **c** Heatmap of top 20 genes from (**b**). **d** Highly involved KEGG and GO biological processes in OPM2 (gray) and Delta47 (red). **e** Oxygen consumption rate (OCR, left) and basal and spare respiration (right) of FOXM1^KO^ (purple) vs FOXM1^KO-R^ (green) OPM2 cells. Addition of metabolic modulators as in Fig. 4e. **f** Same as panel (**e**), except Delta47 cells were used. **g** Extracellular acidification rate (ECAR, left) and glycolysis and glycolytic capacity (right) of FOXM1^KO^ (purple) vs FOXM1^KO-R^ (green) OPM2 cells. Addition of glucose and inhibitors as in Fig. 4f. **h** Same as panel (**g**), except OPM2 was replaced with Delta47. **i** Mean glucose uptake and mean lactate secretion in FOXM1^KO^ cells (purple) or FOXM1^KO-R^ cells (green). **j** As panel (**e**), but Delta47 instead of OPM2.

To strengthen confidence in the above findings, we added back FOXM1 to the FOXM1^KO^ cells by transfecting them with a constitutively expressed *FOXM1c* cDNA gene. The reconstituted OPM2 and Delta47 cells, designated FOXM1^KO-R^, contained high amounts of FOXM1 protein (Fig. 4a), exhibited involvement of *Hypoxia* by GSEA (Fig. 4b, c) and demonstrated gene expression changes similar to those seen in FOXM1^KO^ vs FOXM1^N^ cells (Fig. 3b, c and results not shown). GO and KEGG analyses implicated *Cell cycle* in OPM2 and Delta47 cells, and KEGG additionally involved *Oxidative phosphorylation* in both cell lines (Fig. 4d). By demonstrating that the reconstituted cells (KO-R) harbored biologically active FOXM1, the findings validated FOXM1^KO-R^ for functional follow-up studies. These revealed that basal and spare respiration capacity were markedly enhanced in FOXM1^KO-R^ cells compared to FOXM1^KO^ cells (Fig. 4e, f). ECAR (Fig. 4g, h), glucose uptake and lactate output (Fig. 4i, j) were also significantly higher in FOXM1^KO-R^ cells than in FOXM1^KO^ cells. In sum, these findings strongly indicated that FOXM1 promotes myeloma cell glycolysis and energy production.

### FOXM1 inhibitor, NB73, phenocopies genetic loss of FOXM1 in myeloma

**Fig. 5:**
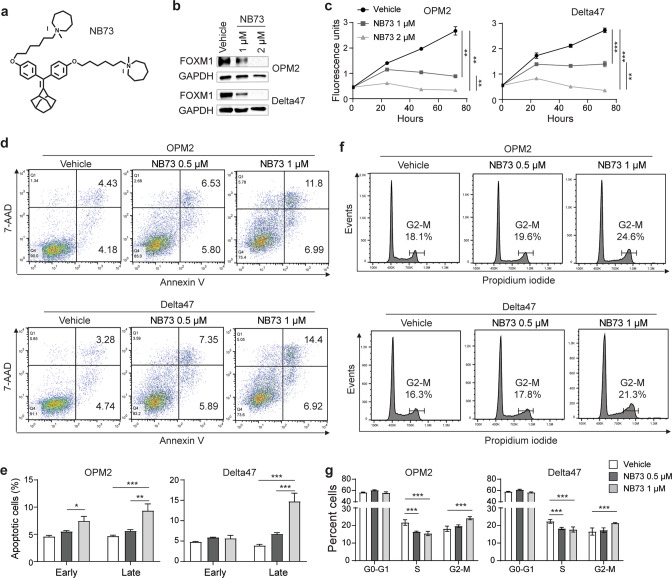
FOXM1 inhibitor, NB73, hampers myeloma growth and survival in vitro. **a** Structural formula of NB73. **b** Western blot of FOXM1 and GAPDH in OPM2 cells (top) and Delta47 cells (bottom) treated for 24 h with the indicated amounts of NB73 (lanes 2–3) or left untreated (lane 1). **c** Growth of OPM2 and Delta47 in cell culture in presence of FOXM1 inhibitor (gray squares and triangles) or solvent (vehicle) control (black circles). **d** Flow cytometric scatter plots of OPM2 (top) and Delta47 (bottom) cells undergoing early (Annexin^+^AAD^-^) and late (Annexin^+^AAD^+^) stages of apoptosis. Cells were treated using the indicated amounts of NB73 (center and right panels) or left untreated (left panels). **e** Mean values (vertical bars) and standard deviations (short horizontal lines) of OPM2 cells (left) and Delta47 cells (right) in early or late apoptosis based on triplicate measurements. **f** Flow cytometry of cell cycle progression of OPM2 (top) and Delta47 (bottom) cells treated with the indicated amounts of NB73 (center and right panels) or left untreated (left panels). Percentage of cells at G2/M is indicated. **g** Mean values and standard deviations of OPM2 and Delta47 cells in three different stages of the cell cycle based on triplicate measurements: G, growth phase; S, synthesis phase, M, mitotic phase.

We evaluated NB73, a newly developed small-drug inhibitor of FOXM1 (Fig. 5a) [10], to facilitate the bench-to-bedside translation of FOXM1-targeted myeloma therapy. NB73 is a 1,1-diarylethylene compound [10] that binds FOXM1 [10] and kills breast cancer cells that contain high amounts of the transcription factor [9]. Treatment of FOXM1^N^ OPM2 and Delta47 cells using 2 μM NB73 abrogated FOXM1 by Western analysis (Fig. 5b). Consistent with that, treatment of these cells with ≤2 μM NB73 hampered cell proliferation (Fig. 5c), aggravated programmed cell death (Fig. 5d, e) and hindered cell cycle progression (Fig. 5f, g). The finding that the IC_50_ of NB73 in myeloma cells that harbor low amounts of FOXM1 (MM1.S and XG-7) was approximately 2-fold higher than that in OPM2 and Delta47 cells (Fig. S7) supports the contention that the inhibitor exhibits specificity. The results described above mimicked the changes seen in FOXM1^KO^ cells left untreated (Fig. 2). In addition, they confirmed published findings on the impact of NB73 on triple negative breast cancer (TNBC) [9] indicating that NB73 is superior to previously developed FOXM1 inhibitors such as FDI-6 [18] and the peptide thiazole antibiotics, thiostrepton [19] and siomycin A [20]. FOXM1-inhibiting antibiotics of this sort demonstrate broad cancer-suppressing activity in the low micromolar concentration range in solid and liquid tumors [21] – including myeloma (Fig. S8) – yet are not suitable for use in humans due to severe toxicity issues. Next, we decided to evaluate whether NB73 may be able to down regulate bioenergetic pathways in myeloma cells. Because our previous work suggested that in order to be fully effective FOXM1 inhibition must be combined with inhibition of other players in the FOXM1 myeloma network [1, 3], we searched for lead compounds that may synergize with NB73 in myeloma. To that end, we took an advantage of the Connectivity Map (CMap) housed at the Broad Institute, Cambridge, MA [22, 23].

### CMap nominates GDA for combination treatments using NB73

**Fig. 6:**
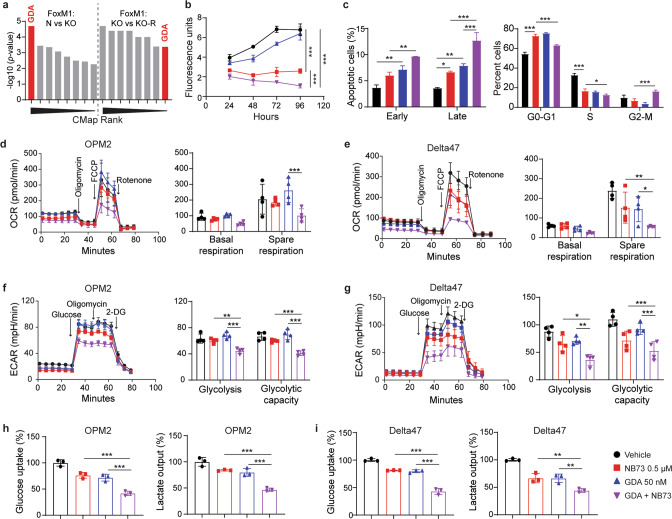
GDA enhances myeloma-inhibiting activity of NB73. **a** CMap nominated drugs and compounds rank ordered according to *p* values in Tables S5 and S6. GDA, highlighted in red, was the only drug revealed by both analyses, taking the 1^st^ rank in the FOXM1^N^ vs FOXM1^KO^ OPM2 dataset (left half) and the 8th rank in the FOXM1^KO^ vs FOXM1^KO-R^ OPM2 dataset (right half). **b** Growth of OPM2 cells treated with 0.5 µM NB73 (red), 50 nM GDA (blue) or both drugs at the same concentration (purple). Cells treated with solvent only (vehicle) were used as control (black). **c** Early and late apoptosis (left panel) and cell cycle progression (right panel) in the 4 groups of OPM2 cells shown in panel (**b**). Both parameters were determined using flow cytometry. **d** Oxygen consumption rate (left) and basal and spare respiration (right) in the 4 groups of OPM2 cells shown in panels (**b**) and (**c**). **e** Same as panel (**d**), except Delta47 was used. **f** Extracellular acidification rate (left) and glycolysis and glycolytic capacity (right) in 4 groups of OPM2 cells. **g** Same as panel f, except Delta47 was used. **h** Mean glucose uptake (left) and mean lactate secretion (right) in in 4 groups of OPM2 cells. **i** Same as panel h, except Delta47 was used.

CMap is a multifunctional online tool that enables users to compare gene expression signatures that arise from genetic perturbations in cancer cells (e.g., loss of FOXM1) to a large library of gene signatures that reflect the impact of small-drug inhibitors on cancer cells. We hypothesized that perturbational drug signatures in the CMap database that are statistically “connected” to (i.e., significantly correlated with) the unique gene signature of FOXM1 deficiency in myeloma may nominate drug candidates that mimic the genetic loss of FOXM1 in myeloma. Candidates of this sort would be expected to cooperate with NB73 in inhibiting myeloma. CMap analysis of DEGs in FOXM1^N^ vs FOXM1^KO^ cells (Fig. 4a) and FOXM1^KO^ vs FOXM1^KO-R^ cells (not shown) uncovered just one drug nominated in both datasets: geldanamycin (GDA, Fig. 6a and Tables S5 and S6). Figure 6b shows that a low dose of GDA (50 nM) was mildly inhibitory in myeloma in vitro. However, in combination with NB73 (0.5 μM), GDA was potent and enhanced NB73-dependent growth inhibition by approximately 50%. Co-treatment using NB73 and GDA was highly effective in triggering myeloma cell apoptosis (Fig. 6c, left) and blocking cell cycle progression (right). Similar results were observed in functional studies on cellular respiration and glycolysis, using the same amounts of drugs used in panels b and c. Thus, the combination of NB73 (0.5 μM) and GDA (50 nM) inhibited mitochondrial respiration in OPM2 (Fig. 6d) and Delta47 cells (Fig. 6e) more effectively than either drug alone. Similarly, cotreatment using NB73 and GDA resulted in stronger inhibition of glycolytic activity in OPM2 (Fig. 6f) and Delta47 cells (Fig. 6g) than treatment with either drug alone. Glucose uptake and lactate production in OPM2 (Fig. 6h) and Delta47 cells (Fig. 6i) were also strongly inhibited by the combined drug administration. These results demonstrated that nanomolar amounts of GDA are well tolerated in myeloma cells and significantly enhance the efficacy with which NB73 inhibits glycolysis and energy production.

### GDA cooperates with NB73 in downregulating FOXM1 in myeloma cells

**Fig. 7:**
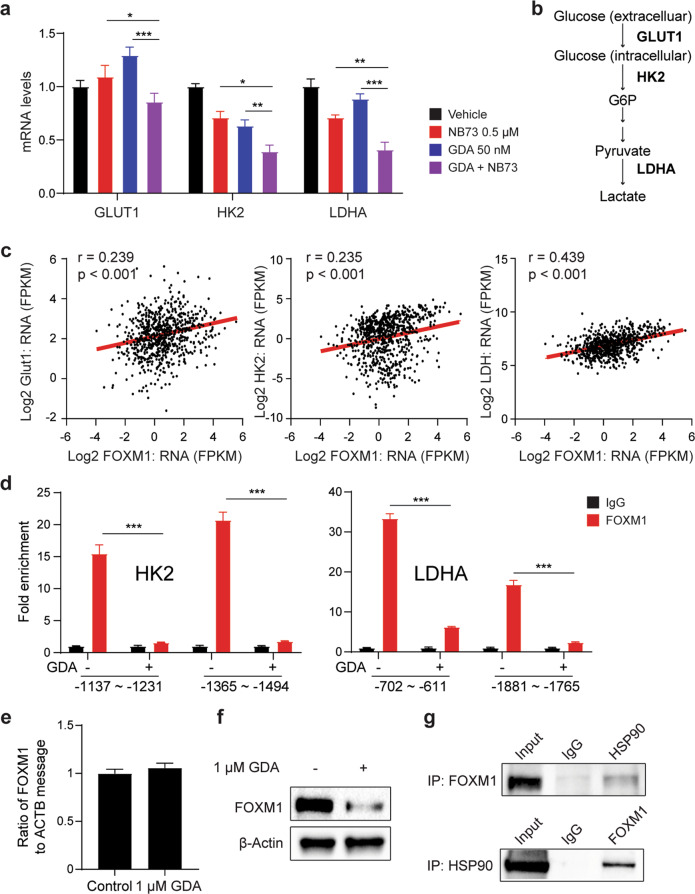
GDA inhibits FOXM1-dependent expression of glycolytic genes. **a** Message levels of 3 glycolytic genes in OPM2 cells exposed for 6 h to 0.5 µM NB73 (red), 50 nM GDA (blue) or both drugs (purple). Cells left untreated were included as control (black). Measurements relied on quantitative RT-PCR. **b** Position of genes from panel a in the metabolic pathway of glycolysis. *GLUT*1 is involved in glucose import. *HK2* and *LDHA* catalyze the first and last step of glycolysis, respectively. **c** Gene expression scatterplots from the MMRF CoMMpass dataset used in Fig. 1. Pearson correlation coefficients are indicated. Linear regression lines have been highlighted in red using Adobe Illustrator. **d** ChIP-PCR analysis of FOXM1 binding to *HK2* and *LDHA* promoter regions in OPM2 cells treated with 50 nM GDA (indicated by “+”) or left untreated (“–"). Size of PCR fragments and their location with respect to transcriptional start sites are indicated below the bar diagram. Fold enrichment of ChIP compared to baseline control was determined with the help of antibody to FOXM1 (red bars) and unrelated antibody of the same isotype (IgG, black bars), respectively. **e** RT-PCR analysis of *FOXM1* mRNA levels in OPM2 cells treated with 1 μM GDA for 24 h or left untreated. **f** Western blot of FOXM1 from samples used in panel e. **g** Co-immunoprecipitation indicating physical interaction of FOXM1 and HSP90 in OPM2 cells. Immunoblots using specific antibodies to HSP90 (after IP using antibody to FOXM1) or FOXM1 (after IP using antibody to HSP90) are shown on top of each other. Isotype controls (labeled “IgG”) and whole-cell lysates not subjected to IP (labeled “input”) were included as controls. Considering that HSP90 interacts with approximately 400 client proteins, the mechanism by which GDA cooperates with NB73 is likely to go beyond the changes found here. In support of that, treatment of cancer cells using the HSP90 inhibitor, PF-4942847, suppressed FOXM1 by an indirect mechanism that involved upregulation of HSP70 [56].

**Fig. 8:**
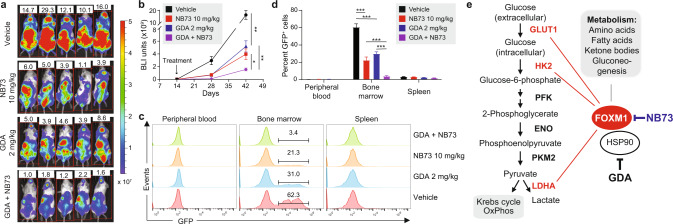
Cotreatment with NB73 and GDA slows down myeloma in NSG mice. **a** BLI images of NSG mice 42 days after tumor challenge (OPM2 cells). Mice were treated, beginning on day 14 after tumor challenge, with indicated drugs. Mice left untreated (top panel) were used as control. BLI intensity (indicated by colored bar to the right) ranged from 0.2–5 × 10^7^ units. **b** Rate of tumor growth in mice from panel a based on quantitative BLI analysis on days 14, 28, and 42 after tumor cell transfer. Mice were treated using the indicated amounts of NB73 (red), GDA (blue) or both drugs (purple). Untreated controls are indicated in black. **c** Flow cytometric detection of GFP-labeled myeloma cells in mice from panel a. Cells of this sort were too rare to be enumerated in peripheral blood and spleen. The percentage of these cells in the bone marrow is indicated. **d** Statistical comparison of the abundance of GFP-positive myeloma cells in the bone marrow of mice from panel (**a**). **e** Working model on the role of FOXM1 in glycolytic energy production in myeloma. This study has shown that GLUT1, HK2 and LDHA are transcriptionally controlled by FOXM1 (indicated by thin red lines) and thus potentially targetable by FOXM1 inhibition for treatment of MM. Preclinical evidence gathered by other investigators has nominated PFK, ENO and PKM2 (bold black) as additional molecular targets for anti-glycolytic therapy of myeloma. This may be significant because activation of glycolysis promotes extra-medullary disease in patients with myeloma [57]. NB73, a small-molecule FOXM1 inhibitor, curbs glycolysis in myeloma. NB73’s efficacy can be enhanced by geldanamycin (GDA), an inhibitor of heat shock protein 90 (HSP90) that stabilizes FOXM1 in myeloma. Additional studies are warranted to elucidate the role of FOXM1 in oxidative phosphorylation (OxPhos) [58] and OxPhos-dependent pathways of drug sensitivity in myeloma [59], and to evaluate whether OxPhos inhibitors, such as "(IACS-010759) [60], under clinical development for cancer therapy [61] are effective in myeloma [62]. An important research task going forward is the mechanism with which FOXM1 impacts the metabolic pathways denoted in the gray text box on the upper right.

We examined whether the co-treatment of myeloma cells using NB73 and GDA suppresses gene expression in the glycolytic pathway. qPCR analysis of FOXM1^N^ OPM2 cells treated with the same amount of drug used in Fig. 7 showed that this was the case for *GLUT1* (a.k.a. solute carrier family 2 member 1, or SLC2A1), *HK2* (hexokinase 2) and *LDHA* (lactate dehydrogenase A, Fig. 7a)—key regulators of glycolysis important for transporting glucose inside the cell, phosphorylating glucose at the C6 position, and converting pyruvate to lactate, respectively (Fig. 7b). Delta47 yielded the same result (data not shown). In contrast, *GLUT4* and *LDHB* message was not affected by NB73 and GDA in either cell line (data not shown). These results suggested that FOXM1 governs, in part, the transcriptional regulation of glycolysis. In support of that, *FOXM1* mRNA levels in the MMRF dataset presented in Fig. 1 correlated with expression of *GLUT1*, *HK2* and *LDHA* (Fig. 7c). Next, we wondered why the co-treatment of OPM2 using NB73 and GDA was more potent in down regulating *HK2* and *LDHA* than *GLUT1* (Fig. 8a). ChIP-PCR analysis suggested that this is related to the efficacy with which GDA removes FOXM1 from the promoter regions of *HK2* and *LDHA* (Fig. 7d) but not *GLUT1* (result not shown). The mechanism with which GDA displaces FOXM1 from gene promoters does not appear to involve in transcriptional downregulation of *FOXM1* (Fig. 7e). Instead, GDA acts at the post-translational level (Fig. 7f), presumably by promoting the proteasomal degradation of FOXM1 due to abrogated HSP90 chaperone function, as seen in leukemia [24]. In agreement with that, co-immunoprecipitation followed by Western blotting, relying on either FOXM1 (upper panel) or HSP90 (lower panel) as bait, produced matching results (Fig. 7g). In sum, these findings demonstrate that in myeloma cells FOXM1 regulates glycolysis at the transcriptional level. Furthermore, HSP90 inhibitor GDA cooperates with NB73 by destabilizing FOXM1 in myeloma.

### NB73 and GDA synergize in inhibiting myeloma-in-mouse xenografts

To complement the in vitro drug studies described above with results using intact laboratory animals, we transfected parental FOXM1^N^ OPM2 cells with a bicistronic lentiviral expression vector that encodes GFP (green fluorescence protein) and Luc (luciferase) reporter genes. The reporters enabled the determination of myeloma cell growth and dissemination in host mice, using flow cytometry (GFP) and BLI (Luc) as research tools. GFP^+^Luc^+^ OPM2 cells were injected IV into immunodeficient NSG mice to generate human-in-mouse xenografts (*n* = 20). Tumor-bearing mice were randomly divided into 4 equal groups (*n* = 5) and, beginning on day 14 after tumor inoculation, treated twice weekly with NB73 (10 mg/kg SC), GDA (2 mg/kg IP) or both. One group of mice was left untreated and used as control. BLI on days 28 and 42 after tumor challenge demonstrated both the therapeutic efficacy of monotherapy using NB73 or GDA and the additive effect of combination therapy (Fig. 8a, b). At study end on day 42, mice were euthanized, and spleen, peripheral blood and bone marrow samples were analyzed for GFP^+^ tumor cells using flow cytometry. Cells of this sort were not detected in peripheral blood but did occur in spleen (≤2%) and, more abundantly, in bone marrow (≤50%, Fig. 8c). The latter agreed with the proclivity of FOXM1^N^ OPM2 cells to home to the bone marrow (Fig. 3d). Figure 8d demonstrates the striking myeloma-inhibiting activity of co-treatment using NB73 and GDA. The synergy between FOXM1 inhibition (NB73) and HSP90 inhibition (GDA) suggests a new treatment approach for patients with myeloma expressing ample amounts of FOXM1.

## Discussion

The main finding of this study is the demonstration that (1) FOXM1 prognosticates inferior survival of patients with myeloma in the MMRF CoMMpass trial (NCT01454297); (2) FOXM1 is a positive regulator of myeloma metabolism with major impact on glycolysis and cellular respiration; (3) the novel small-compound FOXM1 inhibitor, NB73, slows down myeloma; (4) the heat shock protein 90 (HSP90) inhibitor, GDA, cooperates with NB73 in down regulating FOXM1 and suppressing myeloma. The cooperativity of NB73 and GDA suggests that combined FOXM1 and HSP90 inhibition might be a new treatment option for FOXM1-driven HRMM and RRMM, two critical unmet needs in myeloma. NB73 and GDA are included in the working model on the role of FOXM1 in myeloma metabolism presented in Fig. 8e.

Our results on FOXM1-dependent activation of myeloma metabolism agree with evidence that metabolic reprogramming, a hallmark of human cancer, is important for MM [25]. Compared to normal long-lived plasma cells, which exhibit a high rate of glucose consumption [26] due to a high physiological demand for antibody glycosylation [27], neoplastic plasma cells feature further increases in metabolic activity [28] together with characteristics of metabolic remodeling that are typical of the Warburg effect: aerobic glycolysis, elevated glucose consumption, and increased lactate production [29]. FDG-PET imaging takes advantage of these changes for both myeloma patient care and translational myeloma research using laboratory mice [30]. Myeloma cells can also make use of the reverse Warburg effect; i.e., taking up lactate from the tumor microenvironment via the monocarboxyl transporter 1 (MCT1) and utilizing it as substrate for mitochondrial energy production [31]. The role of FOXM1 in this pathway, if any, is not known. Upregulation of mitochondrial biogenesis [32], CD38-driven mitochondrial trafficking [33], mutations in mitochondrial genes [34] and OxPhos stimulation [35] are also involved in the mechanism with which myeloma adapts metabolically and maintains bioenergetic plasticity. The underlying molecular pathways include protein tyrosine phosphatase 4A3 (PTP4A3) [36], ectonucleotide pyrophosphatase / phosphodiesterase 1 (ENPP1) [37] and, as suggested by the current study, FOXM1. Elucidating these pathways in greater depth may lead to new, metabolically targeted myeloma therapies that include FOXM1 inhibition.

This study adds myeloma to a group of solid neoplasms including ovarian cancer [38] and pancreatic cancer [39] for which FOXM1-driven reprogramming of glucose metabolism and promotion of the Warburg effect have been well documented [40]. Our findings on FOXM1-driven expression of glucose import (GLUT1) and glycolytic pathway genes (HK2, LDHA) in OPM2 and Delta47 cells extend published work on the transcriptional regulation of glycolysis in myeloma and suggest that co-inhibition of FOXM1 and glycolysis affords a new treatment approach (Fig. 8E). In addition to FOXM1, LDHA is upregulated in myeloma via HIF1A (hypoxia inducible factor 1 subunit alpha) [41] and PPARGC1B (peroxisome proliferator-activated receptor gamma coactivator 1 beta) [16]. PPARGC1A, the alpha isoform of this coactivator, which is included in both heatmaps in Fig. 3C, may also promote LDHA expression, but this has not been demonstrated. Other investigators have gathered preclinical evidence that targeting glycolytic enzymes can suppress myeloma. Thus, silencing PKM2, a pyruvate kinase muscle isozyme that is positively regulated by the Myc-NEK2 axis in myeloma cells [42], inhibits myeloma [43]. In sync with that, FOXM1-dependent upregulation of PKM2 promotes glycolysis and the Warburg effect in colon cancer [44]. HK2, an isoform of hexokinase that is overexpressed in many malignancies including MM [45], is another promising target in myeloma given that small-drug inhibition of HK2 [46] or knockdown of *HK2* using anti-sense oligonucleotides [47] kills neoplastic plasma cells with great efficacy. Dampening glucose import using the HIV protease inhibitor ritonavir [48] and inhibiting PFK (phosphofructokinase) [49] or ENO (enolase) [50] have also been evaluated as experimental myeloma therapy, albeit with limited success and not in the context of upregulated FOXM1.

This is the first study to demonstrate that NB73 is active in myeloma. NB73 is a small-drug FOXM1 inhibitor from the 1,1-diarylethylene class of compounds [10] that effectively kills breast cancer cells containing high amounts of FOXM1 [9]. Time-resolved fluorescence resonance energy transfer (FRET) has shown that NB73 binds directly to FOXM1 [10] and thus facilitates its proteasomal degradation. NB73 exhibits suitable pharmacokinetic properties, affords an adequate therapeutic window, and is well tolerated in tumor-bearing mice. What is more, NB73 compares favorably to previously developed FOXM1 inhibitors including FDI-6 [18] and RCM-1 [51], which for a variety of reasons are not acceptable for clinical use [13]. This renders NB73 attractive for further development as a cancer drug. Pharmacological inhibition of HSP90 enhanced the potency with which NB73 hinders myeloma. GDA was used here to that end despite its difficult track record in cancer therapy [52] including myeloma [53] that can be attributed to detrimental toxicity due to GDA’s interference with the chaperone activity of all (paninhibition) HSP90 isoforms [54]. Newly developed isoform-selective or C-terminus-targeted HSP90 inhibitors may overcome this problem [52]. An additional, alternative strategy for making FOXM1-targeted myeloma inhibition more effective may be afforded by compounds that feature high CMap enrichment scores in Fig. 5a. Compounds of this sort that also boast a positive track record in preclinical myeloma research include lycorine, quinidine and thapsigargin (Table S5) and semustine, ciclopirox and pyrvinium (Table S6). Future studies will reveal whether any of these compounds synergize with NB73 in hampering FOXM1-driven myeloma.

In conclusion, this study has provided clear-cut evidence that FOXM1 governs myeloma metabolism by upregulating glycolysis and bioenergy supply. Additionally, it has shown that the small-compound FOXM1 inhibitor, NB73, suppresses myeloma by virtue of promoting FOXM1 degradation. This may be of value for targeted therapy of chr1q-amp myeloma that depends on a novel PBX1-FOXM1 axis described just recently [55]. Enhancement of FOXM1 degradation by GDA suggests that co-inhibition of FOXM1 and HSP90 may be a promising precision medicine approach to treating myeloma that contains elevated levels of FOXM1.

## Materials and methods

### Human myeloma cell lines (HMCLs)

HMCLs were obtained from Dr. Brian Van Ness, University of Minnesota, and propagated under standard cell culture conditions (37 °C, 5% CO_2_) in RPMI1640 (Gibco) supplemented with 10% heat inactivated (65 °C, 30 min) fetal bovine serum (Gibco) and 1% v/v antibiotic/antimycotic solution (Gibco). To detect and treat contamination with mycoplasma, the eMyco VALiD Mycoplasma PCR Detection Kit (Bulldog Bio, NH, USA) and Plasmocure (InvivoGen, CA, USA) were used, respectively, as needed. Cell lines were authenticated with assistance of the Human STR Profiling Cell Authentication Service (ATCC, VA, USA).

### CRISPR/Cas9 knockout and isolation of individual clones

Three CRISPR-modified synthetic single guide RNAs (gRNAs) were purchased from Synthego (CA, USA) to target FOXM1 exon 2. Guide RNA was complexed individually with Cas9 protein (a kind gift from Dr. Miles Pufall, Department of Biochemistry, University of Iowa) to make ribonucleoprotein (RNP). For transfection with RNP, myeloma cells were washed and pelleted, followed by gentle resuspension in Nucleofector Solution SF supplemented with Lonza solution and IDT electroporation enhancer buffer. RNP complex was added, and cells were electroporated using a Lonza 4D-Nucleofector in CM-138 mode. Individual cell clones were obtained by limited dilution from the batch edited cells and screened for loss of FOXM1 using Western blotting and Sanger sequencing. Guide RNA sequence and Sanger sequencing primer are listed in Table S7.

### Overexpression of FOXM1

A gene expression vector encoding FOXM1c on a pCDNA3.1 backbone was purchased from GenScript (NJ, USA). The gene was cloned into lentiviral vector pMK1115 expressing GFP. The vector was packaged into viral particles using the Mirus 293 T Trans-IT kit according to the protocol. Myeloma cells were transfected by spinfection, allowed to recover for a week, and then fractionated based on GFP expression using a cell sorter.

### Myeloma xenografting

NSG mice (strain number 005557) were purchased from The Jackson Laboratory, Bar Harbor, Maine and housed according to the rules and regulations of the Medical College of Wisconsin Biomedical Resource Center under IRB AUA6541. Equal numbers of male and female mice 8–10 weeks of age were used. Statistical methods for estimating the sample size were not used. Mice were randomly allocated to experimental groups. For tumor growth studies, host mice were challenged intravenously with 1 × 10^6^ OPM2 cells transfected with eGFP and luciferase reporter genes. For drug treatment studies, 2 × 10^6^ cells were used. Two weeks later, tumor-bearing mice were treated twice weekly with subcutaneous injections of NB73 (10 mg/kg), intraperitoneal injections of GDA (2 mg/kg) or both agents. Tumor burden was measured using the IVIS Spectrum CT in vivo imaging system (PerkinElmer). Humane endpoints included paraplegia and weight loss of more than 10%. The study was not blinded.

### Statistical analysis

All data were analyzed using GraphPad Prism and presented as means ± standard deviation (SD) unless otherwise indicated. For statistical comparison of 2 groups, two-tailed Student’s *t* test was used. For comparison of multiple groups, one-way ANOVA followed by Dunnett posttest was used. A *p* value of <0.05 was considered significant. Sample sizes were determined empirically to ensure sufficient statistical power. No sample was excluded from analysis. Variance between different groups was similar. Results are based on independent biological experiments conducted in triplicate.

### Supplemental materials and method

Additional details on materials and methods used for this study are presented online in the supplemental section of the manuscript.

## Supplementary information

Supplements
