# Supplementary material for: FOXM1 regulates glycolysis and energy production in multiple myeloma
Source: Oncogene. 2022 Jul 6;41(32):3899–911. doi: 10.1038/s41388-022-02398-4 (PMC9355869; doi:10.1038/s41388-022-02398-4)
Supplement: Supplementary file 1 — Supplements [file 41388_2022_2398_MOESM1_ESM.pdf]

## Materials and methods

### Human myeloma cell lines (HMCLs)

HMCLs were propagated under standard cell culture conditions (37 °C, 5% $CO_2$) in RPMI1640 (11875093, Gibco) supplemented with 10% heat inactivated (65 °C, 30 min) fetal bovine serum (10437-028, Gibco) and 1% v/v antibiotic/antimycotic solution (15240062, Gibco). To detect and treat contamination with mycoplasma, the eMyco VALiD Mycoplasma PCR Detection Kit (25231, Bulldog Bio) and Plasmocure (InvivoGen) were used, respectively, as needed. Cell lines were authenticated with assistance of the Human STR Profiling Cell Authentication Service (ATCC, Manassas, VA).

### CRISPR/Cas9 knockout and isolation of individual clones

Three CRISPR-modified synthetic single guide RNAs (gRNAs) were purchased from Synthego to target FOXM1 exon 2. Guide RNA was complexed individually with Cas9 protein (a kind gift from Dr. Miles Pufall, Department of Biochemistry, University of Iowa) to make ribonucleoprotein (RNP). For transfection with RNP, myeloma cells were washed and pelleted, followed by gentle resuspension in Nucleofector Solution SF supplemented with Lonza solution and IDT electroporation enhancer buffer. RNP complex was added, and cells were electroporated using a Lonza 4D-Nucleofector in CM-138 mode. Individual cell clones were obtained by limited dilution from the batch edited cells and screened for loss of FOXM1 using Western blotting and Sanger sequencing. Guide RNA sequence and Sanger sequencing primer are listed in **Table S7**.

### Overexpression of FOXM1

A gene expression vector encoding FOXM1c on a pCDNA3.1 backbone was purchased from GenScript. The gene was cloned into lentiviral vector pMK1115 expressing GFP. The vector was packaged into viral particles using the Mirus 293T Trans-IT kit according to the protocol. Myeloma cells were transfected by spinfection, allowed to recover for a week, and then fractionated based on GFP expression using a cell sorter.

### Western blotting

Cells were processed using the Cell and Tissue Extraction Kit (K269-500, BioVision). Protein concentrations were determined using the Pierce™ BCA Protein Assay Kit (23225, Thermo). Whole cell lysates were incubated with SDS loading buffer (Bio-Rad) and boiled for 5 minutes.

30 μg of protein was fractionated by electrophoresis in 10% SDS-polyacrylamide gels (Bio-Rad), transferred to nitrocellulose filters, probed with antibody, and detected by enhanced chemiluminescence (Bio-Rad). Antibody to FOXM1 (5436, Cell Signaling Technology) and β-Actin (3700S, Cell Signaling Technology) were used.

**Cell viability, apoptosis, cell cycle progression, and clonogenic growth**

PrestoBlue™ Cell Viability Reagent (A13261, Thermo) was used to determine cell viability in vitro. Programmed cell death was analyzed by flow cytometric detection of AnnexinV-APC and 7-AAD (R37176, Invitrogen). Cell cycle progression was measured with the help of the Tali™ Cell Cycle Kit (A10798, Thermo) that relies on propidium iodide (PI) and flow cytometry to differentiate between cells in G0/G1, S and G2/M phase. Soft agar clonogenicity assays were performed as previously described. Briefly, $1.5 \times 10^4$ cells were plated, and colonies were counted after incubation for 4 to 8 weeks.

**RNA sequencing (RNA-seq) and gene set enrichment analysis (GSEA)**

Total RNA was extracted using the RNeasy Plus mini kit (74134, Qiagen). RNA concentration and purity was determined on NanoDrop. RNA sequencing and bioinformatics analysis were performed at BGI Genomics. The RNA-seq data in this article have been deposited in NCBI's Gene Expression Omnibus and is accessible with the accession number: GSE180018.

**Quantitative reverse-transcription PCR (qPCR)**

cDNA synthesis relied on the iScript cDNA Synthesis Kit (1708891, Bio-Rad). Quantitative real-time PCR was performed on the CFX Connect Real-Time System (Bio-Rad) using reagents of iQ SYBR Green SuperMix (1708880, Bio-Rad). Primer sequences are listed in **Table S7**. mRNA levels were calculated using the $2^{-\Delta\Delta Ct}$ method and normalized to ACTB.

**Oxygen consumption rate (OCR) and extracellular acidification rate (ECAR)**

OCR and ECAR were measured on a Seahorse XF96 Extracellular Flux Analyzer (Agilent). Briefly, myeloma cells were suspended in sodium bicarbonate-free RPMI1640 (103576-100, Agilent) supplemented with 10 mM glucose (103577-100, Agilent), 2 mM L-glutamine (25030-081, Gibco) and 1 mM sodium pyruvate (11360-070, Gibco) for the mito stress test, or 2 mM L-glutamine only for the glycolysis stress test. Cells were seeded in Cell-Tak (BD Biosciences) pre-coated XF96 plates at $2 \times 10^5$ cells/well. Plates were spun at 200 × g without breaking and incubated in the XF incubator without $CO_2$ for 30 min to ensure cell attachment. Measurements

were taken prior to addition of inhibitor (basal level) and after sequential injection of 1 μM oligomycin, 0.5 μM FCCP and 1 μM rotenone for mito stress test, or 25 mM glucose, 1 μM oligomycin and 250 mM 2-deocyglucosee for glycolysis stress test.

**Glucose utilization and lactate production**

Cells were seeded into 6-well plates at 1 × $10^6$ per well. Culture medium was collected after 2 days and filtered through a 10 kDa MW spin filter (88513, Pierce™). Glucose utilization (K686-100, BioVision) and lactate production (K627-100, BioVision) were measured in accordance with BioVision protocols.

**Co-immunoprecipitation**

Bio-Rad SureBeads Protein A (1614013, Bio-Rad) or Protein G (1614023, Bio-Rad) Magnetic Beads were incubated for 1 hour with 7.5 μg antibody to HSP90 (37-9400, Invitrogen) or FOXM1 (20459, Cell Signaling Technology). After thorough washing in PBST, 1 mg whole-cell lysate was added and incubated at 4 °C overnight with slow rotation. Proteins were eluted by incubating the beads for 5 min at 90 °C with 20 μL 2× Laemmli Buffer (Bio-Rad). Eluted protein and input extract were analyzed using Western blotting.

**Chromatin immunoprecipitation assay**

The Pierce™ Magnetic ChIP kit (26157, Thermo) was used. Briefly, cells were crosslinked using 1% formaldehyde at room temperature for 10 min and then mixed with glycine for 5 min. After washing twice with ice cold PBS, cells were collected and resuspended in 200 μL membrane extraction buffer containing protease/phosphatase inhibitors, followed by incubation on ice for 10 min. Supernatant was removed and nuclei were resuspended in 200 μL digestion buffer supplemented with micrococcal nuclease (MNase) and incubated at 37 °C for 15 min. DNA was sheared by sonication on ice. Supernatant was incubated with antibody to FOXM1 or isotype control IgG at 4 °C overnight. Immunoprecipitated DNA was retrieved from beads using IP elution buffer containing proteinase K (65 °C, 1.5 hrs). DNA was purified using a DNA clean-up column followed by PCR analysis. Primer sequences are given in **Table S7**.

**Myeloma xenografting**

NOD.Cg-Prkdcscid Il2rgtm1Wjl/SzJ (NSG) mice were purchased from The Jackson Laboratory (strain number 005557, Bar Harbor, Maine) and housed according to the rules and regulations of the Biomedical Resource Center, Medical College of Wisconsin (IRB approval number

AUA6541). Mice of age 8-10 weeks (half female half male) were used in this study. No statistical methods were used to estimate the sample size in this study. Animals were randomly allocated to different groups considering gender and body weight. For tumor growth studies, host mice were challenged intravenously with 1 ×106 OPM2 cells transfected with eGFP and luciferase reporter genes. For drug treatment studies, 2×106 cells were used. Two weeks later, tumor-bearing mice were treated twice weekly with subcutaneous injections of NB73 (10 mg/kg), intraperitoneal injections of GDA (2 mg/kg) or both agents. Tumor burden was measured using the IVIS Spectrum CT preclinical in vivo imaging system (PerkinElmer). Humane endpoints included paraplegia and weight loss of more than 10%. There was no blinding in this study.

**Statistical analysis**

All data were analyzed using GraphPad Prism and presented as means ± standard deviation (SD) unless otherwise indicated. For statistical comparison of 2 groups, two-tailed Student’s t test was used. For comparison of multiple groups, one-way ANOVA followed by Dunnett posttest was used. A p value of < 0.05 was considered significant. Sample sizes were determined empirically to ensure sufficient statistical power. No samples were excluded from analysis. All data represent multiple independent experiments conducted in triplicate. The variance is similar between the groups that are being statistically compared.

**Supplemental Table 1.** Cox proportional hazard analysis of the impact of *FOXM1* expression on overall survival (OS) in 762 patients with myeloma in the MMRF CoMMpass (IA15) cohort

| *FOXM1* [1] | Hazard Ratio [2] | 95% CI [2] | *p* value [2] | Hazard Ratio [3] | 95% CI [3] | *p* value [3] |
|---|---|---|---|---|---|---|
| Q1 | 1.13 | 1.06 – 1.20 | <0.001 | 1.13 | 1.06 – 1.20 | <0.001 |
| Q2 | 2.19 | 1.03 – 4.64 | 0.042 | 2.21 | 1.04 – 4.72 | 0.040 |
| Q3 | 1.27 | 0.56 – 2.90 | 0.564 | 1.29 | 0.56 – 2.96 | 0.544 |
| Q4 | 3.09 | 1.40 – 6.82 | 0.005 | 3.13 | 1.41 – 6.97 | 0.005 |

[1] *FOXM1* mRNA levels in bone marrow plasma cells were considered as a continuous variable in the Cox model. The MMRF CoMMpass cohort (IA15) contains 773 patients; 11 had to be eliminated from the analysis due to missing values. Patients with complete information (n = 762) were divided into 4 equal groups (Q1-4) in accordance with increasing *FOXM1* expression.

[2] Adjusted for clinical parameters including age of patient, ISS stage, M-protein levels, and cytogenetic aberrations in myeloma cells.

[3] Additionally adjusted for *MYC* expression in bone marrow plasma cells.

## Supplemental Table 2: GSEA-revealed MSigDb hallmark pathways (v 7.5.1) in myeloma

| OPM2 | FOXM1 KO vs FOXM1 N | NES | *p* | FDR |
|---|---|---|---|---|
| | **Positively enriched gene sets in N** | | | |
| 1 | UNFOLDED PROTEIN RESPONSE | 1.5 | 0.01 | 0.052 |
| 2 | DNA REPAIR | 1.41 | 0.011 | 0.09 |
| | **Negatively enriched gene sets in N** | | | |
| 1 | EPITHELIAL MESENCHYMAL TRANSITION | -1.860 | 0.000 | 0.007 |
| 2 | UV RESPONSE DN | -1.760 | 0.000 | 0.010 |
| 3 | IL2 STAT5 SIGNALING | -1.650 | 0.002 | 0.023 |
| 4 | APOPTOSIS | -1.550 | 0.000 | 0.045 |
| 5 | ESTROGEN RESPONSE EARLY | -1.450 | 0.008 | 0.104 |
| 6 | INFLAMMATORY RESPONSE | -1.400 | 0.014 | 0.139 |
| 7 | ANGIOGENESIS | -1.390 | 0.091 | 0.134 |
| 8 | MYOGENESIS | -1.360 | 0.020 | 0.134 |
| 9 | KRAS SIGNALING UP | -1.350 | 0.021 | 0.128 |
| 10 | TNFA SIGNALING VIA NFKB | -1.340 | 0.017 | 0.127 |
| **Delta47** | **FOXM1 KO vs FOXM1 N** | | | |
| | **Positively enriched gene sets in N** | | | |
| 1 | KRAS SIGNALING DN | 1.37 | 0.016 | 0.421 |
| 2 | APICAL JUNCTION | 1.35 | 0.017 | 0.252 |
| | **Negatively enriched gene sets in N** | | | |
| 1 | UV RESPONSE DN | -1.850 | 0.000 | 0.002 |
| 2 | KRAS SIGNALING UP | -1.730 | 0.000 | 0.009 |
| 3 | ESTROGEN RESPONSE EARLY | -1.690 | 0.000 | 0.010 |
| 4 | TNFA SIGNALING VIA NFKB | -1.620 | 0.000 | 0.019 |
| 5 | EPITHELIAL MESENCHYMAL TRANSITION | -1.580 | 0.000 | 0.025 |
| 6 | MITOTIC SPINDLE | -1.570 | 0.000 | 0.022 |
| 7 | HEDGEHOG SIGNALING | -1.520 | 0.017 | 0.034 |
| 8 | G2M CHECKPOINT | -1.510 | 0.000 | 0.036 |
| 9 | **HYPOXIA** | -1.470 | **0.004** | 0.052 |
| 10 | COAGULATION | -1.440 | 0.022 | 0.067 |
| **OPM2** | **FOXM1 KOR vs FOXM1 KO** | | | |
| | **Positively enriched gene sets in KO** | | | |
| 1 | MTORC1 SIGNALING | 1.520 | 0.000 | 0.207 |
| 2 | INTERFERON ALPHA RESPONSE | 1.440 | 0.019 | 0.204 |
| 3 | IL2 STAT5 SIGNALING | 1.430 | 0.007 | 0.150 |
| 4 | G2M CHECKPOINT | 1.390 | 0.011 | 0.161 |
| 5 | **HYPOXIA** | 1.380 | **0.007** | 0.139 |
| 6 | TNFA SIGNALING VIA NFKB | 1.370 | 0.011 | 0.128 |
| 7 | IL6 JAK STAT3 SIGNALING | 1.350 | 0.042 | 0.129 |
| 8 | INFLAMMATORY RESPONSE | 1.350 | 0.013 | 0.115 |
| 9 | EPITHELIAL MESENCHYMAL TRANSITION | 1.250 | 0.043 | 0.215 |
| | **Negatively enriched gene sets in KO** | | | |
| 1 | APICAL JUNCTION | -1.39 | 0.01 | 0.293 |
| 2 | ANDROGEN RESPONSE | -1.33 | 0.066 | 0.282 |
| 3 | ESTROGEN RESPONSE LATE | -1.27 | 0.045 | 0.35 |
| **Delta47** | **FOXM1 KOR vs FOXM1 KO** | | | |
| | **Positively enriched gene sets in KO** | | | |
| 1 | OXIDATIVE PHOSPHORYLATION | 2.110 | 0.000 | 0.000 |
| 2 | KRAS SIGNALING UP | 1.580 | 0.000 | 0.047 |
| 3 | G2M CHECKPOINT | 1.580 | 0.000 | 0.032 |
| 4 | ALLOGRAFT REJECTION | 1.580 | 0.000 | 0.025 |
| 5 | IL6 JAK STAT3 SIGNALING | 1.420 | 0.026 | 0.094 |
| 6 | **HYPOXIA** | 1.420 | **0.010** | 0.083 |
| 7 | MYC TARGETS V1 | 1.400 | 0.008 | 0.082 |
| | **Negatively enriched gene sets in KO** | | | |
| 1 | WNT BETA CATENIN SIGNALING | -1.670 | 0.008 | 0.017 |
| 2 | MYOGENESIS | -1.560 | 0.000 | 0.034 |
| 3 | ANGIOGENESIS | -1.530 | 0.024 | 0.035 |
| 4 | APICAL JUNCTION | -1.490 | 0.003 | 0.040 |

## Supplemental Table 3: FOXM1-regulated KEGG pathways in myeloma

| Input: Down regulated genes in FOXM1$^{KO}$ vs FOXM1$^{N}$ OPM2 cells | |
|---|---|
| **Pathway** | *p* value |
| Axon guidance | 0.000 |
| Cell cycle | 0.001 |
| **Oxidative phosphorylation** | 0.002 |
| Cholesterol metabolism | 0.003 |
| Platelet activation | 0.003 |
| B cell receptor signaling pathway | 0.004 |
| Cytokine-cytokine receptor interaction | 0.009 |
| Regulation of actin cytoskeleton | 0.010 |
| Glycolysis / Gluconeogenesis | 0.011 |
| Fc epsilon RI signaling pathway | 0.011 |
| Tryptophan metabolism | 0.013 |
| Hematopoietic cell lineage | 0.017 |
| Pathways in cancer | 0.019 |
| T cell receptor signaling pathway | 0.021 |
| Protein digestion and absorption | 0.031 |
| Neurotrophin signaling pathway | 0.032 |
| Calcium signaling pathway | 0.032 |
| Sphingolipid metabolism | 0.039 |
| Focal adhesion | 0.039 |
| Rheumatoid arthritis | 0.041 |

| Input: Down regulated genes in FOXM1$^{KO}$ vs FOXM1$^{N}$ Delta47 cells | |
|---|---|
| **Pathway** | *p* value |
| Ribosome | 1.58E-13 |
| Alzheimer's disease | 4.76E-12 |
| Thermogenesis | 4.70E-11 |
| Huntington's disease | 3.56E-10 |
| Metabolic pathways | 5.07E-10 |
| Parkinson's disease | 4.76E-08 |
| **Oxidative phosphorylation** | 2.11E-07 |
| Protein processing in endoplasmic reticulum | 2.43E-07 |
| Pathways in cancer | 4.54E-07 |
| Biosynthesis of antibiotics | 1.32E-06 |
| Apoptosis | 3.63E-06 |
| Proteasome | 1.33E-05 |
| Lysosome | 1.33E-05 |
| Hepatitis B | 1.38E-05 |
| Non-alcoholic fatty liver disease (NAFLD) | 2.29E-05 |
| Biosynthesis of secondary metabolites | 6.27E-05 |
| Small cell lung cancer | 8.51E-05 |
| Cell cycle | 8.88E-05 |
| RNA transport | 2.84E-04 |
| Microbial metabolism in diverse environments | 4.44E-04 |

| Input: Up regulated genes in FOXM1$^{KOR}$ vs FOXM1$^{KO}$ OPM2 cells | |
|---|---|
| **Pathway** | *p* value |
| Systemic lupus erythematosus | 2.41E-07 |
| Alcoholism | 5.51E-07 |
| Viral carcinogenesis | 1.26E-03 |
| Sphingolipid metabolism | 7.38E-03 |
| **Oxidative phosphorylation** | 9.37E-03 |
| Cholesterol metabolism | 9.86E-03 |
| Fc epsilon RI signaling pathway | 1.57E-02 |
| Notch signaling pathway | 1.69E-02 |
| Adrenergic signaling in cardiomyocytes | 2.71E-02 |
| Insulin secretion | 3.19E-02 |
| Cell cycle | 3.20E-02 |
| Leukocyte transendothelial migration | 3.86E-02 |
| Necroptosis | 4.52E-02 |
| NOD-like receptor signaling pathway | 4.84E-02 |
| Glycerophospholipid metabolism | 5.39E-02 |
| alpha-Linolenic acid metabolism | 5.50E-02 |
| Th1 and Th2 cell differentiation | 6.05E-02 |
| Linoleic acid metabolism | 6.70E-02 |
| Metabolism of xenobiotics by cytochrome P450 | 7.35E-02 |
| Other types of O-glycan biosynthesis | 7.65E-02 |

| Input: Up regulated genes in FOXM1$^{KOR}$ vs FOXM1$^{KO}$ Delta47 cells | |
|---|---|
| **Pathway** | *p* value |
| Ribosome | 4.19E-35 |
| Thermogenesis | 1.16E-19 |
| Alzheimer's disease | 4.49E-15 |
| **Oxidative phosphorylation** | 7.34E-15 |
| Huntington's disease | 2.03E-14 |
| Parkinson's disease | 3.00E-13 |
| Non-alcoholic fatty liver disease (NAFLD) | 5.92E-13 |
| Spliceosome | 1.16E-10 |
| Protein processing in endoplasmic reticulum | 4.46E-09 |
| RNA transport | 1.10E-06 |
| Proteasome | 1.10E-06 |
| Protein export | 3.42E-06 |
| RNA degradation | 1.04E-05 |
| Cell cycle | 1.15E-04 |
| Longevity regulating pathway | 4.68E-04 |
| Ubiquitin mediated proteolysis | 1.74E-03 |
| Epstein-Barr virus infection | 1.74E-03 |
| Autophagy - yeast | 2.30E-03 |
| Longevity regulating pathway - multiple species | 2.40E-03 |
| Ribosome biogenesis in eukaryotes | 3.96E-03 |

## Supplemental Table 4: FOXM1-dependent GO pathways in myeloma

**Input: Down regulated genes in FOXM1$^{KO}$ vs FOXM1$^{N}$ OPM2 cells**

| **Gene ontology term** | *p* value |
|---|---|
| regulation of biological process | 7.08E-11 |
| regulation of cellular process | 2.59E-10 |
| biological regulation | 3.24E-09 |
| regulation of nitrogen compound metabolic process | 2.05E-08 |
| regulation of cellular metabolic process | 8.29E-08 |
| T cell costimulation | 1.42E-07 |
| regulation of response to stimulus | 5.08E-06 |
| cellular response to oxidative stress | 6.87E-06 |
| fatty acid catabolic process | 1.06E-05 |
| mitotic cell cycle | 1.29E-05 |
| protein-carbohydrate complex assembly | 1.52E-05 |
| regulation of cell adhesion | 1.79E-05 |
| muscle fiber development | 2.36E-05 |
| regulation of cell adhesion | 2.60E-05 |
| regulation of leukocyte activation | 3.12E-05 |
| activation of immune response | 4.78E-05 |
| response to chemical | 6.25E-05 |
| positive regulation of lymphocyte activation | 7.99E-05 |
| response to chemical | 3.25E-03 |
| regulation of response to stress | 6.44E-03 |

**Input: Down regulated genes in FOXM1$^{KO}$ vs FOXM1$^{N}$ Delta47 cells**

| **Gene ontology term** | *p* value |
|---|---|
| cell cycle | 4.45E-15 |
| phosphorylation | 3.23E-14 |
| lipid metabolic process | 5.78E-08 |
| protein transport | 1.25E-06 |
| nuclear-transcribed mRNA catabolic process, nonsense-mediated decay | 4.23E-06 |
| microtubule cytoskeleton organization | 5.81E-06 |
| cell division | 5.81E-06 |
| translational initiation | 7.96E-06 |
| DNA duplex unwinding | 2.12E-05 |
| oxidation-reduction process | 3.23E-05 |
| cell migration | 5.88E-05 |
| neutrophil degranulation | 5.88E-05 |
| immune system process | 7.56E-05 |
| SRP-dependent cotranslational protein targeting to membrane | 7.56E-05 |
| viral process | 7.56E-05 |
| mitotic cytokinesis | 9.57E-05 |
| cellular response to oxidative stress | 1.24E-04 |
| fatty acid metabolic process | 4.47E-04 |
| DNA replication | 4.91E-04 |
| intracellular signal transduction | 4.91E-04 |

**Input: Up regulated genes in FOXM1$^{KOR}$ vs FOXM1$^{KO}$ OPM2 cells**

| **Gene ontology term** | *p* value |
|---|---|
| regulation of lipid transport | 1.08E-18 |
| fat-soluble vitamin catabolic process | 1.78E-12 |
| menaquinone catabolic process | 1.93E-12 |
| cell cycle | 1.93E-11 |
| regulation of binding | 2.73E-11 |
| cardiac cell development | 1.17E-09 |
| regulation of glucose import | 1.74E-09 |
| positive regulation of cholesterol efflux | 2.16E-09 |
| cardiac muscle cell development | 3.12E-09 |
| detection of muscle stretch | 4.75E-09 |
| lymphocyte costimulation | 5.01E-09 |
| regulation of amino acid import | 5.41E-09 |
| cardiac myofibril assembly | 5.75E-09 |
| signal transduction | 5.72E-08 |
| negative regulation of Rac protein signal transduction | 5.86E-08 |
| Notch receptor processing | 6.03E-07 |
| cytokine secretion | 6.14E-07 |
| biological regulation | 6.19E-07 |
| regulation of SMAD protein complex assembly | 6.20E-07 |
| regulation of cellular process | 6.32E-06 |

**Input: Up regulated genes in FOXM1$^{KOR}$ vs FOXM1$^{KO}$ Delta47 cells**

| **Gene ontology term** | *p* value |
|---|---|
| translation | 2.01E-22 |
| cell cycle | 3.77E-20 |
| mRNA processing | 2.89E-19 |
| mRNA splicing, via spliceosome | 1.31E-17 |
| chromatin organization | 1.31E-17 |
| translational initiation | 9.45E-17 |
| RNA splicing | 9.45E-17 |
| SRP-dependent cotranslational protein targeting to membrane | 1.55E-15 |
| nuclear-transcribed mRNA catabolic process, nonsense-mediated decay | 1.27E-13 |
| viral process | 1.77E-13 |
| cell division | 6.40E-12 |
| phosphorylation | 6.61E-12 |
| protein transport | 2.69E-11 |
| mitochondrial translational elongation | 5.53E-11 |
| DNA replication | 4.87E-10 |
| mitochondrial translational termination | 4.93E-10 |
| ribosome biogenesis | 6.82E-09 |
| chromosome segregation | 1.04E-08 |
| rRNA processing | 1.17E-08 |
| RNA metabolic process | 3.29E-08 |

**Supplemental Table 5.** Connectivity Map (CMap) analysis of FOXM1-targeted candidate drugs based on gene expression differences in $FOXM1^N$ vs $FOXM1^{KO}$ myeloma.

| **Rank** [1] | **Drug / compound** [2] | **Mean** [3] | **Anticorrelated** [4] | **n** [5] | **Enrichment** [6] | ***p*** [7] |
|---|---|---|---|---|---|---|
| 1 | geldanamycin | -0.293 | Yes | 15 | -0.577 | 0.00002 |
| 2 | monobenzone | 0.596 | | 4 | 0.873 | 0.00036 |
| 3 | spaglumic acid | 0.779 | | 2 | 0.985 | 0.00044 |
| 4 | lycorine | -0.278 | Yes | 5 | -0.786 | 0.00084 |
| 5 | adrenosterone | 0.590 | | 4 | 0.821 | 0.00179 |
| 6 | natamycin | 0.514 | | 4 | 0.797 | 0.00322 |
| 7 | cinchonine | -0.410 | Yes | 4 | -0.794 | 0.00366 |
| 8 | lisuride | -0.285 | Yes | 5 | -0.698 | 0.00545 |
| 9 | TTNPB | -0.552 | Yes | 2 | -0.948 | 0.00583 |
| 10 | quinidine | -0.417 | Yes | 3 | -0.856 | 0.00587 |
| 11 | ethoxyquin | 0.319 | | 5 | 0.686 | 0.00771 |
| 12 | thapsigargin | -0.374 | Yes | 3 | -0.823 | 0.01104 |
| 13 | ioxaglic acid | 0.544 | | 3 | 0.821 | 0.01144 |
| 14 | 5707885 | -0.231 | Yes | 4 | -0.723 | 0.01204 |
| 15 | atractyloside | -0.383 | Yes | 5 | -0.649 | 0.0131 |
| 16 | ciclopirox | 0.529 | | 4 | 0.706 | 0.01544 |
| 17 | pirenperone | 0.439 | | 5 | 0.643 | 0.01662 |
| 18 | mianserin | -0.411 | Yes | 5 | -0.630 | 0.01782 |
| 19 | moxisylyte | -0.277 | Yes | 5 | -0.626 | 0.01914 |
| 20 | famotidine | -0.247 | Yes | 5 | -0.624 | 0.01949 |

[1] Rank order is based on *p* values in the last column.
[2] From a compendium of ~3,000 drugs for which drug-dependent gene expression changes have been determined.
[3] Average correlation (+) or anti-correlation (-) value.
[4] Only "anti-correlated" drugs are of interest, because drugs that inhibit FOXM1 would be expected to induce gene expression changes that are diametrically opposite to, or "anti-correlated" with, the gene expression signature of FOXM1-proficient myeloma (parental $FOXM1^N$ OPM2 and Delta47 cells) vs FOXM1-deficient myeloma (FOXM1 "knockout" or $FOXM1^{KO}$ OPM2 and Delta47 cells).
[5] Number of datasets.
[6] Enrichment score.
[7] Similarities between the query signature and all signatures in CMap are computed, normalized and converted to a *p* value. This value is used to sort and rank perturbagens (such as drugs) as most similar (correlated) or opposing (anti-correlated). This value was used to generate Figure 7a in the main text.

**Supplemental Table 6.** Connectivity Map (CMap) analysis of FOXM1-targeting candidate drugs based on gene expression differences seen in FOXM1$^{KO}$ vs FOXM1$^{KO-R}$ myeloma.

| **Rank** [1] | **Drug / compound** [2] | **Mean** [3] | **Correlated** [4] | **n** [5] | **Enrichment** [6] | ***p*** [7] |
|---|---|---|---|---|---|---|
| 1 | 5224221 | 0.819 | Yes | 2 | 0.996 | 0.00002 |
| 2 | semustine | 0.666 | Yes | 4 | 0.93 | 0.00004 |
| 3 | phenoxybenzamine | 0.592 | Yes | 4 | 0.919 | 0.00004 |
| 4 | astemizole | 0.656 | Yes | 5 | 0.896 | 0.00004 |
| 5 | ciclopirox | 0.623 | Yes | 4 | 0.9 | 0.0001 |
| 6 | econazole | 0.607 | Yes | 4 | 0.895 | 0.0001 |
| 7 | valinomycin | 0.688 | Yes | 4 | 0.867 | 0.0004 |
| 8 | geldanamycin | 0.311 | Yes | 15 | 0.508 | 0.00042 |
| 9 | ambroxol | -0.366 | | 4 | -0.879 | 0.00052 |
| 10 | tribenoside | 0.595 | Yes | 4 | 0.854 | 0.00062 |
| 11 | scoulerine | 0.513 | Yes | 4 | 0.817 | 0.00205 |
| 12 | 15-delta PGJ2 | 0.311 | Yes | 15 | 0.453 | 0.00226 |
| 13 | equilin | 0.464 | Yes | 5 | 0.749 | 0.00234 |
| 14 | ionomycin | 0.714 | Yes | 3 | 0.883 | 0.0032 |
| 15 | talampicillin | 0.27 | Yes | 4 | 0.794 | 0.00354 |
| 16 | mefloquine | 0.347 | Yes | 5 | 0.717 | 0.00411 |
| 17 | trimethobenzamide | -0.44 | | 5 | -0.715 | 0.00411 |
| 18 | metronidazole | -0.412 | | 5 | -0.712 | 0.00425 |
| 19 | pyrvinium | 0.401 | Yes | 6 | 0.646 | 0.00566 |
| 20 | desoxycortone | 0.229 | Yes | 4 | 0.756 | 0.00674 |

[1-3] As in Supplemental Table 2.
[4] Because drugs that inhibit FOXM1 would be expected to induce gene expression changes that are in sync or correlated with the gene expression signature of FOXM1-deficient myeloma (FOXM1$^{KO}$ OPM2 and Delta47 cells) vs. FOXM1-expressing myeloma (FOXM1 "add back" or reconstituted FOXM1$^{KO-R}$ cells), only "correlated" drugs are of interest here.
[5-7] As in Supplemental Table 2.

**Supplemental Table 7.** Nucleotide sequences (5' → 3') of primers used in this study.

| CRISPR/Cas9 editing (rows 1-3) and verification (row 4) | | |
|---|---|---|
| **Primer name** | **Primer sequence** | |
| Guide RNA 1 | UUGAGAAUCAGUGGCCGACG | |
| Guide RNA 2 | UGAGAAUCAGUGGCCGACGG | |
| Guide RNA 3 | UAAUGAAAACUAGCCCCCGU | |
| Sanger sequencing | AATATTAGCATTGTTGGGGATGGC | |
| Gene expression analysis | | |
| **Primer name** | **Forward** | **Reverse** |
| ACTB | GGGCATGGGTCAGAAGGATT | TCGATGGGGTACTTCAGGGT |
| FOXM1 | AGACCTGTGCAGATGGTGAG | CTGATGGTCTCG AAGGCTCC |
| GLUT1 | CTTTGTGGCCTTCTTTGAAGT | CCACACAGTTGCTCCACAT |
| HK2 | CAAAGTGACAGTGGGTGTGG | GCCAGGTCCTTCACTGTCTC |
| LDHA | ATCTTGACCTACGTGGCTTGGA | CCATACAGGCACACTGGAATCTC |
| ChIP assays | | |
| **Primer name** | **Forward** | **Reverse** |
| HK2-P1 | GTCAGTATTTCATTCTTGCCAG | CTGAGAGGAGTGGAAGCTCCA |
| HK2-P2 | GAAGTTTTGCTGAGAGGCTAG | CTGTCTGCAATGTGTACAGCC |
| LDHA-P1 | CCTGGGTGACAGAGTGAGACC | TCAACCATACCCAAGAACTGT |
| LDHA-P2 | CTGCCCTGAGGTACTCTGAAG | TCCCATCACTCTAGATTCTAAG |

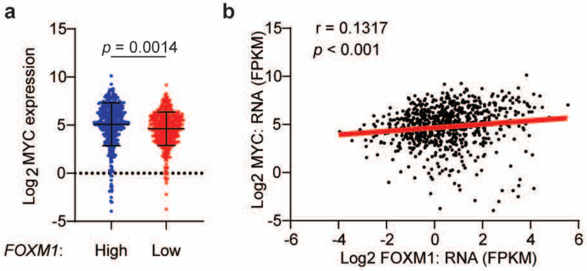


**Coregulation of *MYC* and *FOXM1* expression in myeloma.** Panel **a** shows that *MYC* message levels in the upper half of *FOXM1* expression (5.08 mean, 386 patients) were significantly higher by unpaired two-tailed *t* test than in the lower half of *FOXM1* expression (4.62 mean, 387 patients). But the difference was small: 0.460 +/- 0.143, or 21.2%. Panel **b** depicts the result of a linear correlation analysis of *MYC* and *FOXM1* message in the same dataset (n = 773). There was a modest but significant positive association of gene expression values (Pearson r = 0.1317, $p < 0.001$).

Supplemental Figure 1: Cheng *et al*.

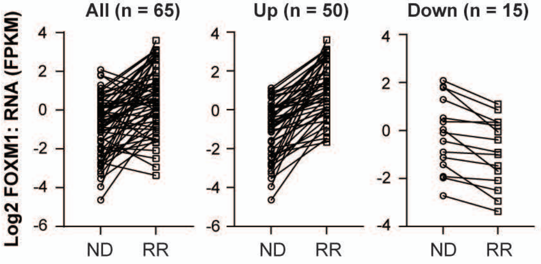


| | | | |
|---|---|---|---|
| *p* value: | <0.001 | <0.0001 | 0.0003 |
| Mean of ND-RR: | 1.45 | 2.10 | -0.725 |
| SD of ND-RR: | 1.83 | 1.55 | 0.595 |
| SEM of ND-RR: | 0.227 | 0.219 | 0.154 |
| 95% CI: | 0.998-1.91 | 1.66-2.54 | -1.05 to -0.395 |
| Pairing: | Effective | Effective | Effective |

***FOXM1* message levels in paired baseline and progression samples from patients with myeloma in the MMRF CoMMpass trial (IA15).** Sixty-five patients were identified for which baseline (ND) and first relapse (RR) gene expression data were available. In 50 cases (77%) *FOXM1* message went up when relapse was compared to new disease. In 15 cases (23%) it was the other way around. Statistical analysis of the entire sample (n = 65, left panel) using a paired *t* test demonstrated that mean *FOXM1* levels in progression samples were on average 2.10 times higher ($1.45^2 = 2.10$) than in baseline samples ($p < 0.001$). The increase was higher (4.41-fold) when the comparison was limited to the 50 cases exhibiting increased gene expression upon relapse (center panel, $2.10^2 = 4.41$, $p < 0.0001$). The decrease in gene expression seen in 15 cases was modest (~50%, $0.725^2 = 0.526$) yet significant ($p = 0.0003$).

Supplemental Figure 2: Cheng *et al*.

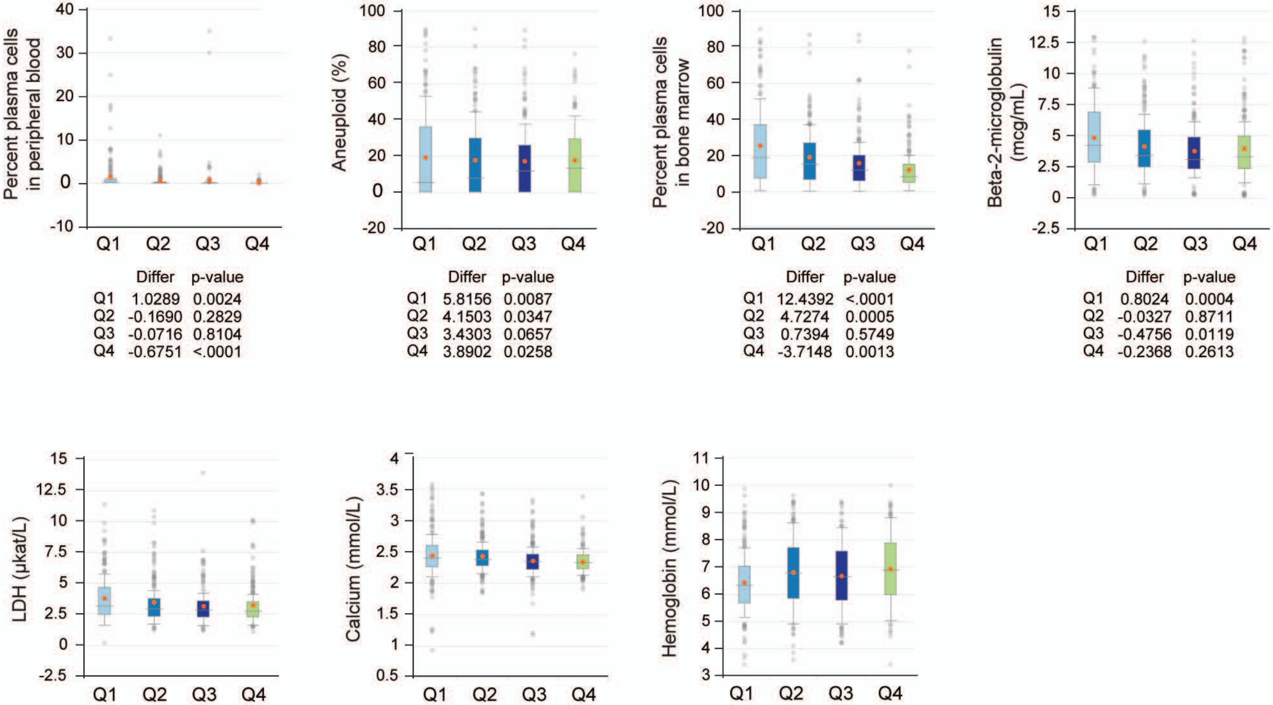


| | Differ | p-value |
|---|---|---|
| Q1 | 1.0289 | 0.0024 |
| Q2 | -0.1690 | 0.2829 |
| Q3 | -0.0716 | 0.8104 |
| Q4 | -0.6751 | <.0001 |

| | Differ | p-value |
|---|---|---|
| Q1 | 5.8156 | 0.0087 |
| Q2 | 4.1503 | 0.0347 |
| Q3 | 3.4303 | 0.0657 |
| Q4 | 3.8902 | 0.0258 |

| | Differ | p-value |
|---|---|---|
| Q1 | 12.4392 | <.0001 |
| Q2 | 4.7274 | 0.0005 |
| Q3 | 0.7394 | 0.5749 |
| Q4 | -3.7148 | 0.0013 |

| | Differ | p-value |
|---|---|---|
| Q1 | 0.8024 | 0.0004 |
| Q2 | -0.0327 | 0.8711 |
| Q3 | -0.4756 | 0.0119 |
| Q4 | -0.2368 | 0.2613 |

| | Differ | p-value |
|---|---|---|
| Q1 | 0.4552 | 0.0040 |
| Q2 | 0.0687 | 0.6627 |
| Q3 | -0.3268 | 0.0148 |
| Q4 | -0.2285 | 0.1175 |

| | Differ | p-value |
|---|---|---|
| Q1 | 0.0720 | 0.0126 |
| Q2 | 0.0621 | 0.0032 |
| Q3 | -0.0275 | 0.2014 |
| Q4 | -0.0488 | 0.0036 |

| | Differ | p-value |
|---|---|---|
| Q1 | -0.3787 | <.0001 |
| Q2 | 0.0686 | 0.4859 |
| Q3 | -0.0875 | 0.3425 |
| Q4 | 0.2120 | 0.0267 |

**Clinical features of myeloma in the MMRF CoMMpass dataset**. Patients were stratified into quartiles based on *FOXM1* mRNA levels. Quartile 1 (Q1) exhibits peak levels. Two-sided *t* testing was used to compare the difference (Differ) of the mean value of one particular quartile (e.g., Q1) with the mean value of the other quartiles (in this case Q2-4). The associated *p* value indicates whether the difference was statistically significant. The order of plots from left to right and top to bottom follows the bar diagram in Figure 1b.

Supplemental Figure 3: Cheng *et al*.

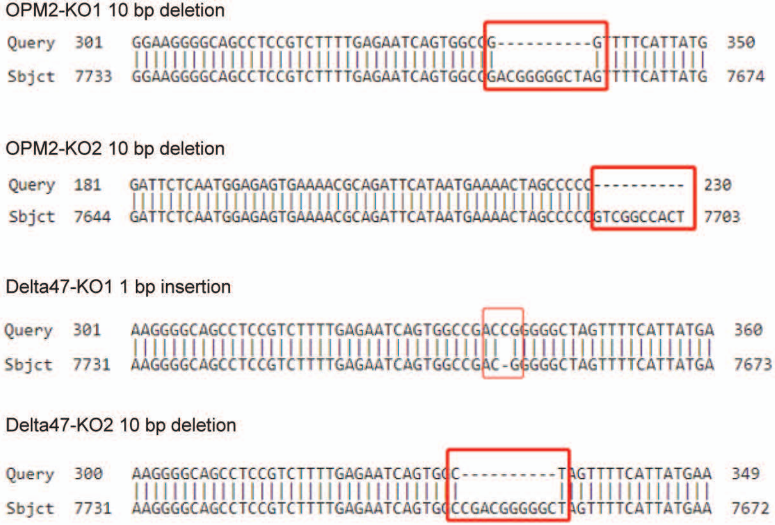


**Gene editing of *FOXM1* in myeloma using CRISPR-Cas9**. Shown are Sanger sequencing results of OPM2 cells (upper half) and Delta47 cells (lower half). In case of OPM2, two independent “knockout” clones, designated KO-1 and KO-2, contain 10-basepair deletions in different sites of the gene (indicated by red boxes). In case of Delta47, KO-1 harbors a 1-basepair insertion that results in a frameshift mutation. KO-2 contains a 10-basepair deletion in close proximity to the one found in OPM2 KO-1.

Supplemental Figure 4: Cheng *et al*.

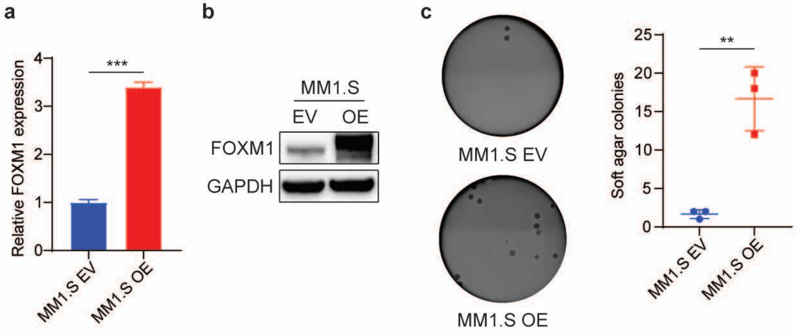


**Forced expression of FOXM1 results in increased clonogenicity of MM1.S myeloma.**
**a** *FOXM1* message measured with the help of qPCR. MM1.S cells were transfected with a bicistronic *FOXM1* and *GFP* (green fluorescent protein) cDNA expression cassette (MM1.S OE) or an "empty vector" that only encoded GFP (MM1.S EV).
**b** Western blot of FOXM1 protein using GAPDH as loading control.
**c** Representative photographic images of soft-agar plates depicting the clonal outgrowth of MM1.S EV and MM1.S OE cells (black dots). The mean number of colonies (long horizontal lines) and standard deviation of the mean (short horizontal lines) were determined in 3 independent experiments.

Supplemental Figure 5: Cheng *et al*.

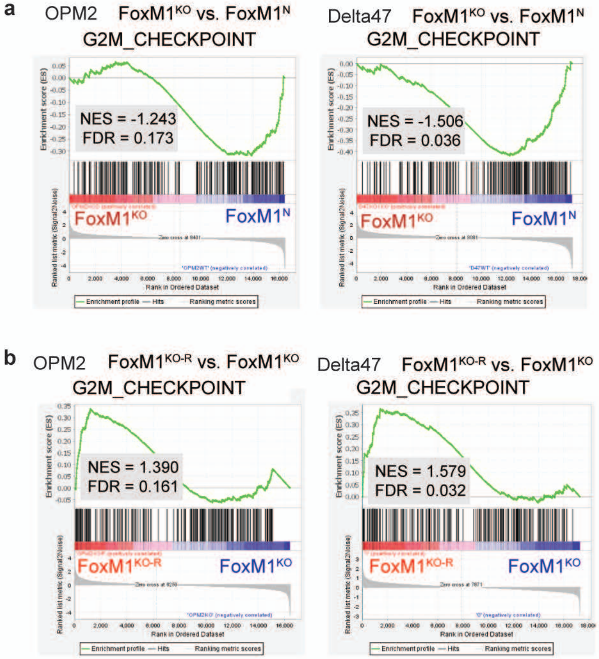


**FOXM1-dependent expression of G2/M checkpoint genes.** Presented are gene set enrichment analysis (GSEA) plots indicating strong activation of the G2/M checkpoint in both $FOXM1^{KO}$ compared to $FOXM1^{N}$ myeloma cells (panel **a**) and $FOXM1^{KO\text{-}R}$ compared to $FOXM1^{KO}$ myeloma cells (panel **b**). $FOXM1^{KO}$, $FOXM1^{N}$ and $FOXM1^{KO\text{-}R}$ cells lack FOXM1 or contain normal and elevated levels of FOXM1, respectively. Normalized enrichment scores (NES) and false discovery rates (FDR) are given in grey text boxes. G2/M checkpoint genes that are upregulated (red) or downregulated (blue) are depicted by thin, vertical, black lines below the enrichment profiles (green curve). OPM2 and Delta47 is shown to the left and right, respectively. Note that enrichment scores in panel a and b are negative and positive, respectively.

Supplemental Figure 6: Cheng *et al*.

| | FOXM1 level | $IC_{50}$ (nM) |
|---|---|---|
| OPM2 | High | 412 |
| Delta47 | High | 505 |
| MM1.S | Low | 1,068 |
| XG-7 | Low | 1,023 |

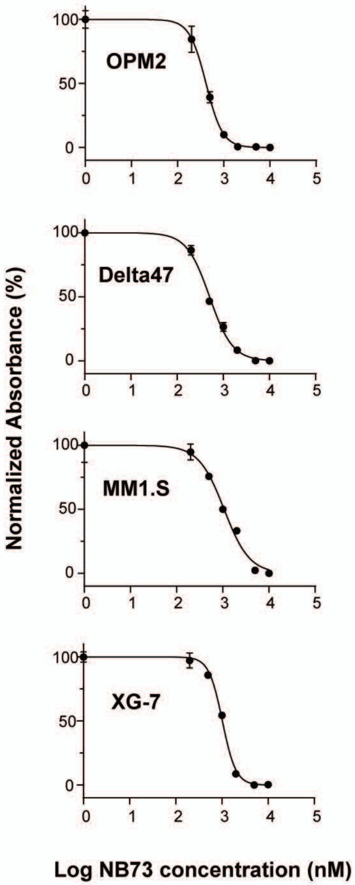


**Growth inhibition of myeloma using FOXM1 inhibitor, NB73.** $FOXM1^{High}$ myeloma (OPM2 and Delta47) was compared to $FOXM1^{Low}$ myeloma (XG-7 and MM1.S). Cell growth was measured with the help of the MTT assay. The half maximal inhibitory concentration ($IC_{50}$) of NB73 was determined using the GraphPad Prism 9 software tool.

Supplemental Figure 7: Cheng *et al*.

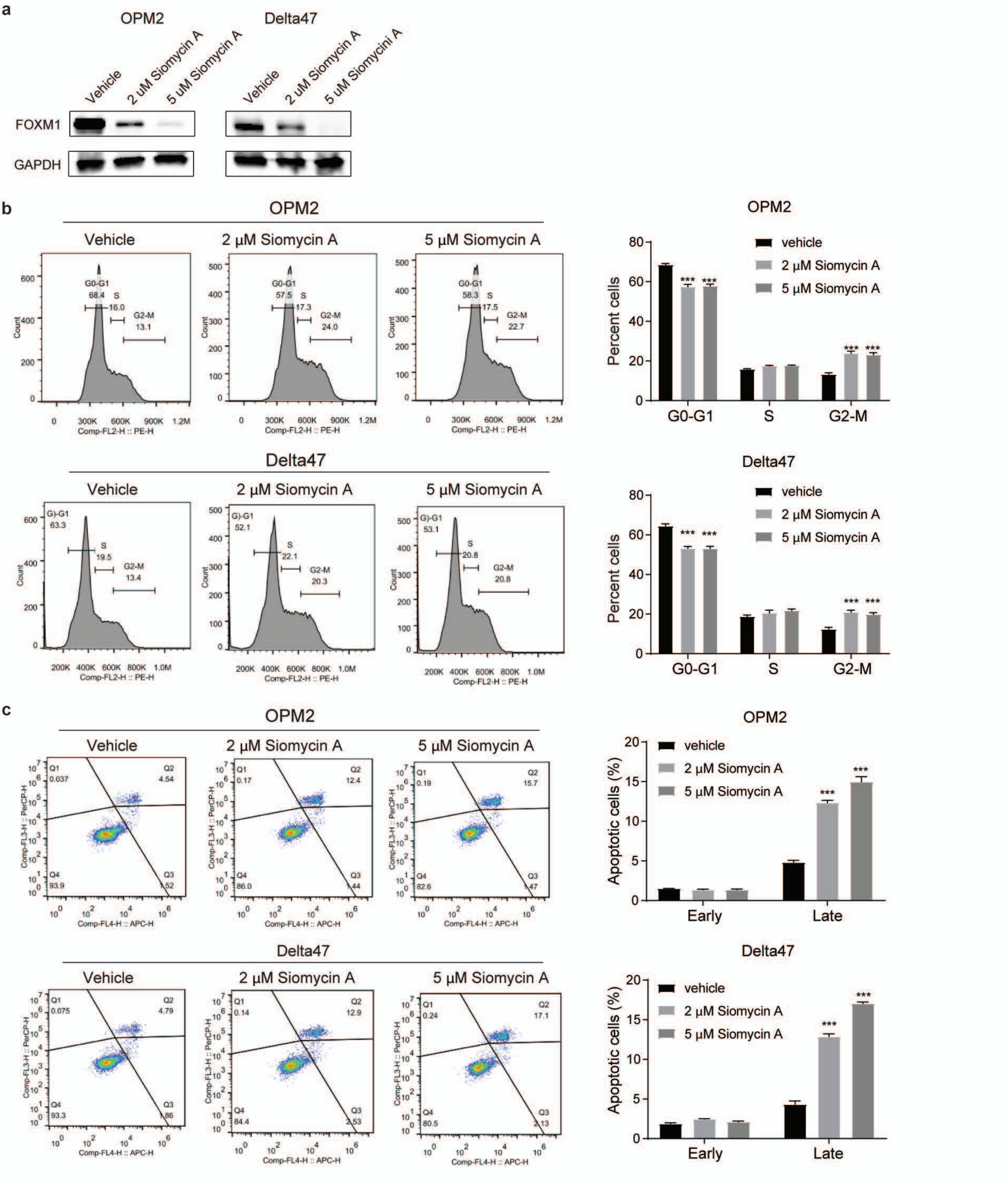


**FOXM1 inhibitor, siomycin A, inhibits myeloma in vitro. a** Western blot of FOXM1 and GAPDH in OPM2 cells (left) and Delta47 cells (right) treated for 24 hours with the indicated amounts of inhibitor (lanes 2-3) or left untreated (lane 1). **b** Flow cytometric determination of cell cycle progression of OPM2 (top) and Delta47 (bottom) treated with siomycin A (center and right panels) or left untreated (left panels). Percentages of cells in different stages of the cell cycle are plotted to the right. **c** Flow cytometric scatter plots of OPM2 (top) and Delta47 (bottom) undergoing early and late stages of apoptosis. Cells were treated using inhibitor (center and right panels) or left untreated (left panels). Mean values (vertical bars) and standard deviations (short horizontal lines) of cells undergoing apoptosis (triplicate measurements) are plotted on the right.
